# Biomedical Probes Based on Inorganic Nanoparticles for Electrochemical and Optical Spectroscopy Applications

**DOI:** 10.3390/s150921427

**Published:** 2015-08-28

**Authors:** Abdulhadee Yakoh, Chanika Pinyorospathum, Weena Siangproh, Orawon Chailapakul

**Affiliations:** 1 Electrochemistry and Optical Spectroscopy Research Unit (EOSRU), Department of Chemistry, Faculty of Science, Chulalongkorn University, 254 Phayathai Road, Patumwan, Bangkok 10330, Thailand; E-Mails: i_style@windowslive.com (A.Y.); n8h7@hotmail.com (C.P.); 2 Department of Chemistry, Faculty of Science, Srinakharinwirot University, Sukhumvit 23, Wattana, Bangkok 10110, Thailand

**Keywords:** biomedical probes, inorganic nanoparticles, electrochemical detection, colorimetry, spectrophotometric detection, biosensors, bioanalysis

## Abstract

Inorganic nanoparticles usually provide novel and unique physical properties as their size approaches nanometer scale dimensions. The unique physical and optical properties of nanoparticles may lead to applications in a variety of areas, including biomedical detection. Therefore, current research is now increasingly focused on the use of the high surface-to-volume ratios of nanoparticles to fabricate superb chemical- or biosensors for various detection applications. This article highlights various kinds of inorganic nanoparticles, including metal nanoparticles, magnetic nanoparticles, nanocomposites, and semiconductor nanoparticles that can be perceived as useful materials for biomedical probes and points to the outstanding results arising from their use in such probes. The progress in the use of inorganic nanoparticle-based electrochemical, colorimetric and spectrophotometric detection in recent applications, especially bioanalysis, and the main functions of inorganic nanoparticles in detection are reviewed. The article begins with a conceptual discussion of nanoparticles according to types, followed by numerous applications to analytes including biomolecules, disease markers, and pharmaceutical substances. Most of the references cited herein, dating from 2010 to 2015, generally mention one or more of the following characteristics: a low detection limit, good signal amplification and simultaneous detection capabilities.

## 1. Introduction

Among the various nanomaterials, inorganic nanoparticles are extremely important in the development of sensors. Not only can they be easily synthesized, but also cheaply mass produced. For this reason, they can also be more readily integrated into a variety of applications. Recently, inorganic nanoparticles of different kinds and dimensions have become widely exploited as versatile and sensitive sensors or probes. The main objective of designer inorganic nanoparticles, enhanced sensitivity in bio-sensing applications, greatly benefits from their small size, where their properties are strongly influenced by increasing their surface area. Thus, the combination of inorganic nanoparticles and sensors is one of the most exciting areas in modern analytical detection development because they offer excellent prospects for designing highly sensitive and selective sensors. Electrochemical sensing and spectrophotometric detection based on the modification or use of a particular variety of inorganic nanoparticles cosntitute a fascinating research area in biomedical applications. The remarkable features resulting from the use of inorganic nanoparticles are now widely employed in various detection systems. Presently, inorganic nanoparticle electrochemical sensors and optical spectrophotometric sensors used for bioanalysis purposes are collected. In this review the electrochemical and optical spectrophotometric characteristics of biomedical probes based on inorganic nanoparticles and the sensing applications possible with these materials are discussed, and their advantages and weaknesses are also explored. We also summarize the promising future anticipated for the use of these inorganic nanoparticles in both electrochemical and spectrophotometric detection as shown in the following sections.

## 2. Types of Nanoparticles

### 2.1. Gold Nanoparticles

Gold nanoparticles (AuNPs), one of the most commonly used metal nanoparticles, have been applied in various fields. The range of applications for AuNPs is growing rapidly and includes: electronics, photodynamics, therapeutic agent delivery, sensors, probes, diagnostics and catalysis. AuNPs possess several advantages: (1) AuNPs can be synthesized in a straightforward manner and can be made highly stable; (2) AuNPs possess unique optoelectronic properties; (3) AuNPs provide high surface-to-volume ratio with excellent biocompatibility; (4) these properties of AuNPs can be readily tuned by varying their size, shape, and the surrounding chemical environment and (5) AuNPs offer a suitable platform for multifunctionalization with a wide range of organic or biological ligands for the selective binding and detection of small molecules and biological targets [1]. Due to the abovementioned advantages, AuNPs have been utilized in various sensing strategies.

### 2.2. Magnetic Nanoparticles

Magnetic nanoparticles are nanoparticles composed of magnetic elements, particularly iron oxide. A large surface-to-volume ratio owing to their small size provides them with a high immobilization density and high surface reactivity [2,3,4]. Prior to their use in any detection procedure, most magnetic nanoparticles have to be functionalized with enzymes, metals, or metal oxides in order to improve their physiochemical properties and stability [5,6]. This refinement has to be employed since bare magnetic nanoparticles tend to form large aggregates and have few active groups. Moreover, they are easily oxidized and dissolved in acid media, leading to poor stability [5]. Their plethora of applications benefit from their low toxicity, biocompatibility, and easy separation utilizing external magnetic fields [7,8,9,10]. A facile separation of magnetic nanoparticles is not only able to reduce the background interference but also enhance target immobilization [11]. Consequently, magnetic nanoparticles have been extensively applied in biomedicine, immunology, biocatalysis, bioanalysis, and especially the separation and purification of target molecules [9,11].

### 2.3. Nanocomposites

Nanocomposites are one of the new composite materials formed by nanometer-sized materials dispersed in a 3-D substrate. Nanocomposites offer an exciting and practical approach for designing and fabricating new technological products and materials with superior mechanical, electrical, optical, antimicrobial, catalytic, and reactive properties [12]. Nanocomposites provide better performance over conventional composite materials and are suitable candidates to overcome the limitations of many materials owing to the high surface to volume ratio of the reinforcing phase and its high aspect ratio. Nanocomposite materials can be classified, according to their matrix materials, into three different categories including ceramic matrix nanocomposites (CMNC), metal matrix nanocomposites (MMNC) and polymer matrix nanocomposites (PMNC) [13]. Herein, to the best of our knowledge, all the various applications of nanocomposites in the biomedical field are presented.

### 2.4. Semiconductor Nanostructures

Semiconductor nanostructures have recently attracted considerable attention due to their unique physical properties that give rise to many potential applications. Semiconductor particles of nanometer size can refer to nanoparticles, nanoclusters, nanocrystals, quantum dots, *etc.* Semiconductor nanostructures are generally categorized into three types: two-dimensional (2D) nanostructures (e.g., quantum wells (QWs)), one-dimensional (1D) nanostructures (quantum wires (QWRs)) and nanowires (NWs)), and zero-dimensional (0D) nanostructures (quantum dots (QDs)) [14]. Colloidally synthesized semiconductor nanoparticles often possess a strong band-gap luminescence tunable by size as a result of the quantum confinement effect, which makes them interesting for different applications [15]. Because QDs are zero-dimensional, they have a sharper state density than higher-dimensional structures and as a result, they have superior transport and optical properties and are being researched for use in laser diodes, solar cells, and several biological sensors [14].

### 2.5. Silver Nanoparticles

Silver nanoparticles have received tremendous attention due to their unique properties that are absent from their bulk forms. Their unusual features, including physiochemical and electronic properties, lead to enormous sensing applications in biosensors, electronics, catalysis, pharmaceutical, and therapeutics [16,17]. Enhancement of the local electric field supports the use of silver nanoparticles as substrates for surface-enhanced Raman scattering (SERs) extending the various uses of silver nanoparticles as detection probes [18]. Silver nanoparticles, moreover, are applied as a sensor modification in order to increase the electrochemical sensitivity, selectivity, and reproducibility [19]. In addition, the aggregation of silver nanoparticles induced by various conditions causes shifting and broadening of the plasma band [16]. This simplicity has allowed the development of abundant naked eye and optical detection uses. Furthermore, the utilization of silver nanoparticles offers benefits of minimal material consumption and no need for sophisticated instruments [16].

### 2.6. Other Nanoparticles

Other metals such as nickel, copper, palladium, and platinum have been used to synthesize metallic nanoparticles. A variety of applications are proposed elsewhere due to their special properties at the nanoscale. Many metal nanoparticles are candidates for electrochemical applications thanks to their conductivity enhancement and signal amplification properties. In addition to an increment in sensitivity, these nanoparticles are often employed due to their cost-effectiveness and environmental friendliness. Most of these applications focusing on electrochemical techniques are described in this review.

## 3. Biomedical Applications

### 3.1. Amino Acids

Amino acids are biological organic compounds that play critical functions in animals. Thiol-containing amino acids, including cysteine and tryptophan, has been frequently reported as disease biomarkers [20], due to the fact that their deficiency leads to numerous disorders, such as Alzheimer’s disease, slowed growth, lethargy, liver damage, skin lesions, *etc*. [21,22]. A rapid quantification of amino acids, especially sulfur-containing compounds, is consequently an important objective for health diagnosis.

#### 3.1.1. Electrochemical Detection

Gold nanoparticles with carbon nanotubes pre-cast on a glassy carbon electrode (AuNP-CNT/GCE) had been fabricated for the detection of tryptophan in pharmaceutical samples with a low detection limit. The hybrid nanomaterial substantially decreased the overpotential of tryptophan because of a remarkable synergistic effect of the modified electrode on the electrocatalytic activity toward the oxidation of the analyte [23].

Fe_3_O_4_-based nanoparticles with graphene oxide have been cast on glassy carbon electrodes. They show high catalytic effects in the oxidation of amino acids. The composite provided the advantages of excellent catalytic activity, high sensitivity, and good stability. The techniques were able to detect cysteine and N-acetylcysteine at low μM levels [24].

A glassy carbon electrode (GCE) was fabricated with silver nanoparticles/graphene oxide (AgNPs/GO) and glucose as a reducing and stabilizing agent. The modified electrode possessed specific features typical of both silver nanoparticles and graphene oxide. The properties of high specific area and fast electron transfer rate improved the electrocatalytic performance and enhanced the activity for oxidation of tryptophan by ten-fold compared with graphene oxide films. The AgNPs/GO/GCE was also free of interferences from tyrosine and coexisting species [25].

Cobalt and nickel nanoparticle-modified electrodes have also been used for the detection of amino acids [21,22,26]. Essential compounds in living cells of animals could be simultaneously detected by a carbon paste electrode (CPE) modified with a (9,10-dihydro-9,10-ethananoanthracene-11,12-dicarbox-imido)-4-ethylbenzene-1,2-diol (DEDE) and a NiO/CNT nanocomposite. The modified electrode had a potent and persistent electron-mediating behavior and well-separated oxidation peaks of cysteine, nicotinamide adenine dinucleotide (NADH), and folic acid were observed [26]. Another work reported nickel oxide nanoparticles modified on a glassy carbon electrode with DNA as a new platform for entrapment of an osmium (III) complex as an excellent electron transfer mediator. The GC/DNA/NiO_x_NP_s_/O_s_(III)-complex modified electrode exhibited excellent selectivity, electrocatalytic activity, stability, remarkable antifouling properties, and allowed the simultaneous detection of cysteine and homocysteine without interferences from low molecular mass biothiol derivatives and electroactive biological species [21]. Cysteine was electrochemically detected by an electrode modified with cobalt hexacyanoferrate nanoparticles with a core shell structure. In the presence of cysteine, the anodic peak current of the Fe(II)/Fe(III) transition was increased while the corresponding cathodic peak current was decreased. In contrast, the peak current of Co(II)/Co(III) remained almost unchanged. The results indicated that the nanoparticles oxidized cysteine via a surface mediated electrocatalytic mechanism. The detection limits of cysteine in batch and flow mode with this modified electrode were as low as 40 and 20 nM, respectively.

#### 3.1.2. Colorimetric and Spectrophotometric Detection

Carboxymethyl cellulose-functionalized gold nanoparticles were synthesized with sodium carboxymethyl-cellulose (CMC-AuNPs) for cysteine detection based on a colorimetric method. The novel nanoparticles would protect particles against salt-induced aggregation. In the presence of cysteine, colloid solutions in sodium chloride were aggregated and displayed color changes, as well as UV-Vis absorption spectra changes. The method was applied to real urine samples [27]. Biothiols including Cys, GSH, and Hcys were detected by a colorimetric assay. *S*-adenosyl-l-methionine (SAM) that interacted electrostatically with unmodified gold nanoparticles (AuNPs) induced selective aggregation. In the presence of bioethics, AuNPs prefer to react with thiols rather than SAM due to the formation of AuS bonds, thus the aggregation of AuNPs changes to the disperse state. The color change was detected by UV-Vis spectrometry and by the naked eye. This assay exhibited rapid operation, high selectivity and sensitivity, and allowd the simultaneous detection of three biothiols compound [28].

Magnetic particles were functionalized with amine and Ni^2+^ for the detection of histidine. A highly specific interaction between the histidine and Ni^2+^ formed a complex, which in the presence of formic acid would liberate gaseous nickel tetracarbonyl which separated from the sample matrix. The gas was determined by atomic absorption/fluorescence spectrometry. Ten to hundred fold improvements over conventional methods was seen with the method. The approach promises high sensitivity, simple design, and convenient operation [29].

Fluorescence responses of Hcy, l-cys, and GSH of CdTe QDs using l-cysteine as capping reagent were investigated. The probe offered good sensitivity, and selectivity for detecting Hcy, l-cys, and GSH in the presence of 20 amino acids, metal ions, and other molecules in biological fluids. It was applied in four serum samples, cell extract sample from two cancer cell lines (Hela and HepG2) with a detection limit of 46 nM, 43 nM, and 63 nM for Hcy, l-cys, and GSH, respectively [30]. Mono-6-SH-β-cyclodextrin capped Mn-doped ZnS quantum dots (β-CD-MnZnS QDs) had dual photoluminescence (PL) at 430 and 598 nm upon excitation at 315 nm to detect tryptophan enantiomers differently. The D-isomer showed little effect whereas L-tryptophan displayed a large time-dependent enhancement in the PL intensity of QDs. This selectivity is due to different inclusion constants for tryptophan enantiomers of the coating on the surface of Mn-ZnS QDs. l-Tryptophan can be detected in the presence of its stereoisomer with a 5.4 nM detection limit [31].

Silver nanoparticles has been primarily reported for detection of amino acids due to the strong interaction of thiol groups towards nanoparticles [32]. Silver nanoparticles induced an aggregation or an anti-aggregation mechanism in the presence of cations and surface modified material. This basis was used for sensing various amino acids. For example, in the presence of cysteine, the color of the silver nanoparticles changed from yellow to pink. The color change was detected visually and could be estimated metrically by measuring the surface plasmon resonance absorption. The method could be applied for the detection of cysteine at ultralow levels [32]. Moreover, nonionic fluorosurfactant-capped silver nanoparticles were aggregated as a result of the presence of cysteine. The modified nanoparticles exhibited selectivity towards cysteine in the determination of this amino acid in human urine and plasma samples [16]. Calcium ion was used as a cross-linking agent for a rapid detection of cysteine. By monitoring the color change from yellow to red, cysteine could be quantified in biological fluids, such as serum and artificial cerebrospinal fluid [33]. Another demonstration stated that cysteine was selectively detected in the presence of Ca^2+^ and NaCl. The result showed other amino acids had no effect on the color change due to the absence of thiol groups [34]. Chromium ion had a similar interaction. In a solution composed of silver nanoparticles and Cr^3+^, cysteine was able to induce aggregation and displayed accolor change from yellow to purple. The techniques also exhibited the selectivity towards cysteine in the presence of other amino acids [35]. Amino acids without thiol functional groups such as tryptophan could be quantified using surface plasmon absorption. The modification of 4,4-bipyridine-functionalized onto silver nanoparticles changed the color of the solutions from yellow to red. The absorption could be detected at 390 nm and 556 nm, respectively. This approach demonstrated the selectivity of the functionalized nanoparticles over other neutral amino acids with low detection limits [36].

### 3.2. Antigen-Antibody

Antigens are many substances that stimulate the immune system to produce antibodies. There is a specific antibody for each antigen, thus, each one can be used to detect the presence of the other. The detection of particular antigens/antibodies is a widespread method used in medical diagnostics of many diseases. Several different methods may be employed, including the following:

#### 3.2.1. Electrochemical Detection

**Figure 1:**
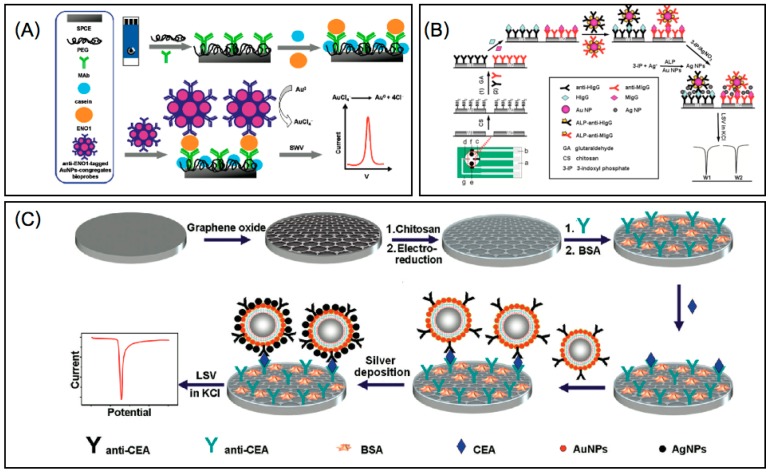
Schematic representation of (**A**) the operation of the electrochemical immunosensor for the detection of ENO1 [37]. Reprinted with permission from (Ho, J.A.A.; Chang, H.C.; Shih, N.Y.; Wu, L.C.; Chang, Y.F.; Chen, C.C.; Chou, C. Diagnostic detection of human lung cancer-associated antigen using a gold nanoparticle-based electrochemical immunosensor. *Anal. Chem.* **2010**, *82*, 5944–5950.). Copyright (2010) American Chemical Society.; (**B**) preparation of immunosensor array and detection strategy by sandwich-type immunoassay and linear sweep voltammetric stripping analysis of enzymatically deposited AgNPs [38]. Reprinted (adapted) with permission from (Lai, G.; Yan, F.; Wu, J.; Leng, C.; Ju, H. Ultrasensitive multiplexed immunoassay with electrochemical stripping analysis of silver nanoparticles catalytically deposited by gold nanoparticles and enzymatic reaction. *Anal. Chem.* **2011**, *83*, 2726–2732.). Copyright (2011) American Chemical Society; and (**C**) the immunosensor fabrication and sandwich-type immunoassay procedure [39]. Reprinted (adapted) with permission from (Lin, D.; Wu, J.; Wang, M.; Yan, F.; Ju, H., Triple signal amplification of graphene film, polybead carried gold nanoparticles as tracing tag and silver deposition for ultrasensitive electrochemical immunosensing. *Anal. Chem.* **2012**, *84*, 3662–3668.). Copyright (2012) American Chemical Society.

In recent years, gold nanoparticles (AuNPs) have found wide use and have gained much attention in the electrochemical sensor field due to their great properties as described above. Interestingly, an electrochemical immunosensing platform has been developed for the detection of the human lung cancer-associated antigen ENO1, by first fabricating a polyethylene glycol (PEG) layer on a screen printed electrode and subsequently using anti-ENO1-tagged AuNPs congregate bioprobes as signal amplifiers to improve the sensitivity of the assay. The electrochemical signal from the bound AuNPs congregates was obtained after oxidizing, followed by the reduction of AuCl_4_^−^ in square wave voltammetry (SWV) mode as shown in Figure 1A. This AuNPs congregate-based assay provides an amplification approach for detecting ENO1 at trace levels, leading to a detection limit as low as 11.9 fg (equivalent to 5 µL of a 2.38 pg/mL solution) [37]. A number of groups have also proposed a signal amplification strategy for ultrasensitive immunosensors. For example, using human and mouse IgG as model analytes, a multiplexed immunoassay has been developed by combining alkaline phosphatase (ALP)-labeled antibody functionalized AuNPs (ALP-Ab/AuNPs) and enzyme-AuNPs catalyzed deposition of silver nanoparticles (AgNPs) on an immunosensor array (Figure 1B). After sandwich-type immunoreactions, the ALP-Ab/AuNPs were captured on an immunosensor surface to catalyze the hydrolysis of 3-indoxyl phosphate, which produced an indoxyl intermediate to reduce Ag^+^. The silver deposition process was catalyzed by both ALP and AuNPs, which amplified the detection signal. The deposited silver was then measured by anodic stripping analysis in KCl solution [38]. Based on a similar concept, a triple signal amplification strategy was also designed as illustrated in Figure 1C. An enhancement in signal resulted from the use of a graphene modified immunosensor surface which accelerated electron transfer, PSA (poly(styrene-co-acrylic acid) microbeads that carried AuNPs as tracing tags to label signal antibody (Ab_2_) and AuNPs induced silver deposition in KCl solution for anodic stripping analysis [39].

#### 3.2.2. Colorimetric and Spectrophotometric Detection

Nanomaterials have found widespread use for the detection of different antigen/antibody combinations. Various optical immunoassay detection methods are of interest in early diagnostic and screening detection, such as a surface-enhanced Raman scattering (SERS)-based gradient optofluidic sensor developed by Chon *et al.*, for testing a specific target marker (rabbit immunoglobulin (IgG)) [40]. The sensor is composed of three components consisting of the gradient channel, the injection and mixing area of antibody-conjugated hollow gold nanospheres and magnetic beads, and a sandwich immunoassay trapping area. This system, using antibody-conjugated HGNs and magnetic beads, provides advantages over the SERS immunoassay performed in microwells owing to the automatically control of the microfluidic system, so the tedious manual dilution process and time consuming assay was eliminated.

Based on magnetic core/shell Fe_3_O_4_/SiO_2_ and Fe_3_O_4_/Ag/SiO_2_ nanoparticles, a surface plasmon resonance (SPR) biosensor for the detection of goat anti-rabbit IgG was developed. The modified magnetic nanoparticles (MNPs)-based biosensor has two main advantages over traditional biosensors. First, the MNPs can easily be immobilized on the Au film, which greatly simplifies the operation; Second, in contrast to the traditional biosensor that contained only one layer of receptor molecules on the surface of the gold film, the modified MNPs have larger surface areas, better compatibilities and more numbers of receptor molecules, which are more beneficial for the immobilization of antibodies [5].

Nanocomposites have also been used for carcinoembryonic antigen (CEA) detection. A sandwich type electrochemiluminescence (ECL) immunosensor based on a Ag/graphene nanocomposite was designed using nanoporous Pd as a catalytically promoted nanolabel. The main advantages of the developed immunosensor can be attributed to two aspects: first, the obtained Ag/graphene nanocomposite could be an ideal substrate for antibody immobilization with good stability and bioactivity. Second, the novel ECL label of nanoporous Pd exhibited excellent ECL activity [41]. A versatile immunosensor using a CdTe quantum dot (QDs) coated silica nanosphere (Si/QDs) as a label was also developed for the detection of a biomarker (rabbit IgG). In this approach, goat anti-rabbit IgG antibody was covalently bound to CdTe QDs on the surface of silica nanospheres (Si/QD/Ab_2_) attached onto the gold electrode surface through a subsequent sandwich immunoreaction. The resulting immunosensor exhibited a signal amplification in both the ECL and square-wave voltametry techniques which could be attributed to the high loading of CdTe QDs [42]. An electrochemiluminescence (ECL) immunosensor based on the amplifying ECL of luminol by hemin-reduced graphene oxide (hemin-rGO) and silver nanoparticles (AgNPs) decorated reduced graphene oxide (Ag-rGO) was constructed for the detection of carcinoembryonic antigen (CEA). 

In brief, AuNPs electrodeposited (DpAu) onto hemin-rGO constituted the base for the immunosensor, which amplified the ECL signal of luminol and served as carrier to immobilize primary antibody (Ab_1_). Moreover, AgNPs-rGO were used to load secondary antibody (Ab_2_) and GOD. In the presence of oxygen, the loaded GOD immediately catalyzed the oxidation of glucose in the detection of *in situ* generated H_2_O_2_, which could promote the oxidation of luminol with an amplified ECL signal. Additionally, hemin and AgNPs could further enhance the ECL signal of luminol owing to the decomposable catalysis of H_2_O_2_ to produce increased amounts of reactive oxygen species [43].

### 3.3. Antioxidants

Antioxidants are a broad group of organic compounds that are widespread in food [44]. Their properties are understood to involve the inhibition of free radical chain reactions. The reactions initiated by free radicals cause damage to proteins, lipids, and nucleic acids [45]. Glutathione (GSH) as an example of antioxidants prevents aging, cancer, and other diseases [46]. In addition, it functions in protein synthesis, enzyme activity, and cell protection [47]. Another example is ascorbic acid (AA) or vitamin C, which acts as a supplement and is frequently consumed in tablets. Mostly, it is used for treating colds, scurvy, and promoting health development [48]. The benefits of other antioxidants are abundantly reported, therefore, their quantification is of importance.

#### 3.3.1. Electrochemical Detection

Modified nanocomposites are responsible for the improvement of electrochemical signals, as a consequence of the good properties of the various materials and nanoparticles fabricated into an electrode. A *N*-(4-hydroxyphenyl)-3,5-dinitrobenenamide-FePt/CNTs carbon paste electrode was used for the detection of GSH in the presence of piroxican. The modified nanocomposite exhibited a good electron-mediating behavior. As a result, the oxidation peaks were well separated. Moreover, the approach could detect GSH and piroxican at low nM concentrations [46]. The synergistic effect of a Fe_2_O_3_/graphene nanocomposite modified electrode was improved for ascorbic acid and uric acid detection [48]. The nanocomposite modified onto the electrode was able to resolve the overlapping anodic peaks of these two analytes. It had advantages of simplicity, high sensitivity, and good selectivity.

Silver nanoparticles/carboxylated multiwalled carbon nanotubes/polyaniline film (AgNPs/c-MWCNT/PANI) have been synthesized on gold electrodes and further covalently immobilized with glutathione oxidase for the detection of glutathione in hemolysated erythrocytes. The modified electrode held a great promise of stability, lower response time and could perform without interferences [47].

Electrodeposition of nickel oxide nanoparticles onto a glassy carbon electrode offered low oxidation potential toward GSH, while other organic compounds like ascorbic acid, uric acid, dopamine, and glucose had no interaction in the method. Additionally, nickel oxide nanoparticles modified with ethylferrocene (EF) and multiwalled carbon nanotubes (MWCNT) on carbon paste electrode (CPE) showed excellent individual peak separation characteristics for the electroxidation of glutathione and acetaminophen [49]. Other nanoparticles such as platinum nanoparticles had been employed as a nanocomposite for Pt-nanoparticles/polyelectrolyte-functionalized ionic liquid (PFIL)/graphene sheet (GS) modified electrodes. Independent oxidation peaks of ascorbic acid and dopamine were observed in urine samples [50].

#### 3.3.2. Colorimetric and Spectrophotometric Detection

Glutathione was detected based on an anti-aggregation mechanism. The solution of gold nanoparticles changed from a dispersion to at aggregated state in the presence of glutathione, which resulted in a color change from red to blue. This anti-aggregation activity was preferable to have higher selectivity. This approach had selectivity towards glutathione relative to natural amino acids, homocysteine, and glutathione disulfide [51]. Gold nanoclusters coated with Hg^2+^ and Au^+^ were able to quench the emission of NIR fluorescence, and the mechanism was used for the detection of glutathione. In the addition of the analyte, the signal was enhanced as a result of the affinity between glutathione and Hg^2+^. The method had been employed for the detection of glutathione in living cells and human blood samples. It possessed advantages of high sensitivity and low spectral interferences [52].

CdS nanotube (NT) films and K_2_S_2_O_8_ as coreactant were modified on an indium tin oxide substrate. Nanosemiconductors coated on this substrate exhibited strong electrochemilluminescent emission. The quench was differently affected by hydroxyl moieties in the benzene ring. This quenching property was used for the simultaneous determination of phenolic compounds, namely catechol, phenol, hydroquinone, and resorcinol with good reproducibility [53]. CdS-2-mercapto-propionic acid or CdS-2-MPA was used for the luminescent detection of rutin. The signal resulted from the inner filter effect and a statistic luminescence quenching component. Rutin was quantified in this technique without interferences from the flavonoids hesperidin and herperetin [54].

Due to the color change of silver nanoparticles from colorless to yellow caused by ascorbic acid, the method could be used for quantification of this antioxidant by LSPR with low detection limit [55]. Another approach presented the immobilization of silver nanoparticles on the surface of magnetic particles for the detection of glutathione by surface plasmon resonance. In the presence of crystal violet, the glutathione competed for the adsorption on the particles. As a result, the Raman signal was decreased and inversely proportional with the increase of glutathione concentration. This method was applied to blood samples with high sensitivity, selectivity, and stability [56].

### 3.4. Cancer

According to the National Cancer Institute (NCI), a biomarker is a biological molecule found in blood, other body fluids, or tissues that is a sign of a normal or abnormal process, or of a condition or disease. A biomarker may be used to see how well the body responds to a treatment for a disease or condition such as cancer. Cancer biomarkers can include a broad range of biochemical entities, such as nucleic acids, proteins, and small metabolites as well as whole tumor cells that indicate the presence of cancer in the body. Cancer biomarkers can be used to screen for cancer diagnosis. Moreover, recent technological advancements have enabled the examination of many potential biomarkers, providing great opportunities for improving the management of cancer patients.

#### 3.4.1. Electrochemical Detection

Electrochemical detection combined with several nanomaterials offers great potential for the detection of clinically significant cancer biomarkers, and recently, various electrochemical biosensors for the detection of cancer biomarkers have been established. For example, an inkjet-printed gold nanoparticles (AuNPs) array immunosensor was fabricated for the multiple detection of the cancer biomarker interleukin-6 (IL-6) in serum. The AuNPs ink was printed on a flexible, heat resistant polyimide Kapton substrate. Captured antibodies for IL-6 were linked onto the eight electrode array, and used in sandwich immunoassays. In addition, a biotinylated secondary antibody with 16–18 horseradish peroxidase labels was used, and detection was achieved by hydroquinone mediated amperometry. These promising sensors are easily fabricated at relatively low cost, and could be mass-produced with commercial inkjet printers [57]. Furthermore, an electrochemical single nucleotide polymorphism (SNP) genotyping sensor for the analysis of a cancer-related gene sequence has also been reported. Combination of the gold nanoparticles (AuNPs)-base enrichment effect with the surface hybridization-based dragging strategy can improve detection sensitivity and signal amplification. With a large number of ferrocene (Fc) probes enriched with AuNPs and then dragged into close proximity to the electrode surface through DNA hybridization, a detection limit at the femtomolar level was achieved [58].

An aptamer-based competition assay for the electrochemical detection of acute leukemia cells was developed with high sensitivity. It utilized the competitive binding of cell-specific aptamers to acute leukemia cells and subsequent voltammetric quantification of the metal signature. Enhanced sensitivity was achieved with dual signal amplification using Fe_3_O_4_ magnetic nanoparticles (MNPs) as carrier to load a large amount of AuNPs and AuNPs-catalyzed silver deposition [59].

**Figure 2:**
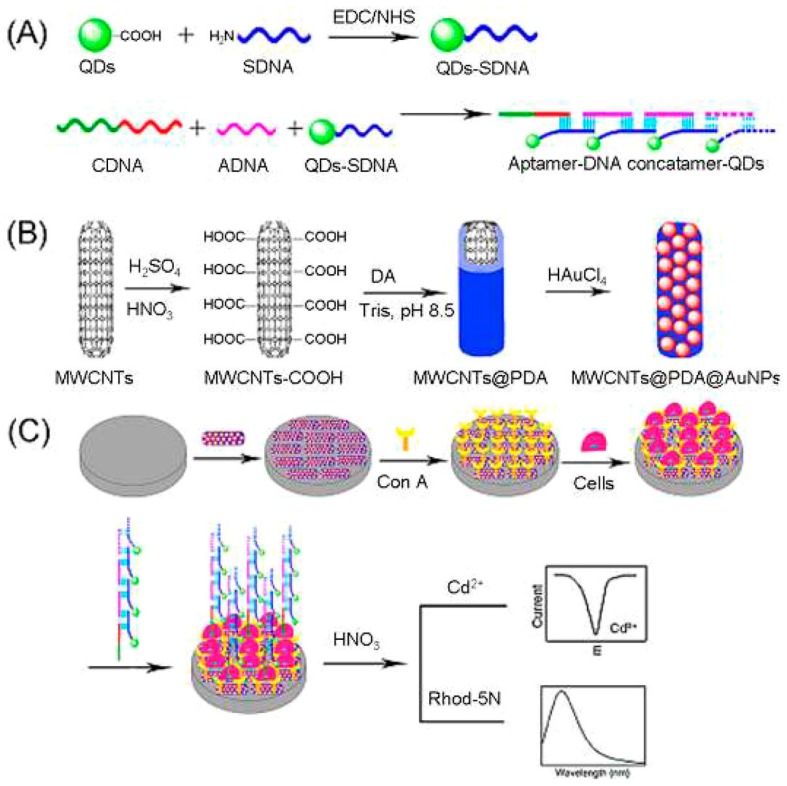
Procedures for the Fabrication of Aptamer-DNA Concatamer-QDs (**A**) MWCNTs@PDA@AuNPs Composites; (**B**) and Supersandwich Cytosensor; (**C**) [60]. Reprinted with permission from (Liu, H.; Xu, S.; He, Z.; Deng, A.; Zhu, J.J. Supersandwich cytosensor for selective and ultrasensitive detection of cancer cells using aptamer-DNA concatamer-quantum dots probes. *Anal. Chem.* **2013**, *85*, 3385–3392.). Copyright (2013) American Chemical Society.

With aptamer-DNA concatamer-quantum dots (QDs) as recognizing probes, model cancer cells (CCRF-CEM cells) were detected using a MWCNTs@PDA@AuNPs modified electrode. The as-prepared electrode was applied to bind concanavalin A (Con A) for cell capture as shown in Figure 2. The developed supersandwich cytosensor showed high sensitivity with a detection limit of 50 cells·mL^−1^. More importantly, it could distinguish cancer cells from normal cells, which indicated the promising applications of the method in the clinical diagnosis and treatment of cancers [60]. Other sensors have recently been demonstrated for the electrochemical detection of specific DNA sequences of bladder cancer cells based on CdTe quantum dots (QDs) modified glassy carbon electrode (GCE). Methylene blue (MB) was intercalated into the hybridized double stranded DNA (dsDNA) and used as electrochemical indicator for the detection of target DNA in a differential pulse voltammetry (DPV) mode. The CdTe QDs provided advantages of excellent electrochemical signal amplification due to their larger surface area, which can immobilize more single stranded DNA (ssDNA) probes on the electrode surface [61].

#### 3.4.2. Colorimetric and Spectrophotometric Detection

Many methods have been used to detect cancer biomarkers, such as a simple colorimetric assay reported by Wang *et al.* [62] for testing human telomerase activity. Telomerase is over-expressed in over 85% of all known human tumors. The working principle is based on the elongated primers conjugated to the gold nanoparticles (AuNPs) surface, which can fold into a G-quadruplex to protect the AuNPs from aggregation. Their assay has also been used for initial screening of telomerase inhibitors as anticancer drug agents. Recently, based on a localized surface plasmon resonance (LSPR) and the coupling plasmon mode of AuNPs, a strategy for the one-step dual detection of tumor-specific mutations (E542K and E545K) and methylation of circulating tumor DNA (ctDNA) of PIK3CA gene has been reported. Peptide nucleic acid (PNA) is used as probe to capture and enrich the 69-bp PIK3CA ctDNA. Immunogold colloids are exploited as methylation detectors and plasmon coupling based enhancement for secondary response. Their results demonstrated that the sensor can simultaneously detect hot-spot mutations and epigenetic changes on ctDNA with this platform [63].

Magnetic nanoparticles (MNPs) have found widespread use for the detection of various cancer biomarkers. For example, the simultaneous detection of two biomarker of T helper cancer cells has been established. One biomarker conjugates with immunofunctionalized MNPs, enabling the separation of the T helper cells from a mixed population of cells. The other biomarker is used for the detection during ELISA analysis. The specific T helper cells can be quantified according to their ELISA absorbance values following magnetic separation [64]. In a similar assay, functionalized MNPs were also utilized for signal enhancement in conjunction with surface plasmon resonance (SPR) on gold nanoslits to detect mRNA heterogeneous nuclear ribonucleoproteins (hnRNP B1) in two cancer cell lines (CL1-0 and CL1-5). In this approach, MNPs were applied for a dual purpose: to isolate the target molecule from the sample matrix to prevent non-specific binding and to enhance the SPR response. The approach for the detection includes double hybridization at two different specific locations in two steps. First, the biomarker target molecules are captured with MNPs, and second, MNPs carrying the target molecules are introduced to the SPR chip to hybridize with probe immobilized on the gold nanoslits [65]. Another work has been reported by Fang *et al.* [66]. In their study, aptamer-conjugated upconversion nanoparticles (UCNPs) was used as nanoprobes to recognize circulating tumor cells (CTC), which were then enriched by attaching magnetic nanoparticles (MNPs) and placing them in the presence of a magnetic field. Owing to the autofluorescence-free nature of upconversion luminescence imaging, as well as the use of maganetic separation to further reduce background signals this method shows promise for CTC detection in medical diagnostics. Up to date, ELISA have most widely been applied in immunoassays. However, several unavoidable limitations of natural enzymes have hindered their widespread applications. Interestingly, an efficient colorimetric detection of target cells utilizing the superior catalytic activity of graphene oxide-magnetic-platinum nanohybrids has been presented. The nanohybrids consisted of Fe_3_O_4_ magnetic nanoparticles (MNPs) and platinum nanoparticles (PtNPs), simultaneously immobilized on the surface of graphene oxide (GO). Due to the highly catalytically active PtNPs and MNPs on GO whose frameworks possess high substrate affinity, the nanohybrid is able to achieve up to 30-fold higher maximal reaction velocity (V-max) compared to that of free GO for the colorimetric reaction of the peroxidase substrate, and enable rapid detection of target cancer cells. Specifically, using this assay system, breast cancer cells can be detected in 5 min with high specificity and sensitivity [67].

Detection of cancer biomarkers using semiconductor quantum dots (QDs) is another promising area of the research. Highly enhanced electrochemilunescence (ECL) from a novel hybrid gold/silica/CdSE-CdS quantum dots nanostructures has been establised for the first time, and successfully applied to develop an ultrasensitive ECL immunosensor for the detection of a protein tumor marker [68]. Liu *et al.* [69] also reported the use of multiplexed QDs and wavelength-resolved imaging to detect and characterize a class of low-abundant tumor cells in Hodgkin’s lymphoma. To overcome the cellular heterogeneity and rarity problem, they have developed multicolor QD antibody conjugates to simultaneously detect a panel of four protein biomarkers (CD15, CD30, CD45, and Pax5) directly in human tissue biopsies. Furthermore, a versatile electrochemiluminescence (ECL) assay for cancer cells based on dendrimer/CdSE-ZnS-quantum dot nanoclusters (NCs) as ECL probe has been reported. Capture DNA was designed as a high affinity aptamer to the target cell; a novel ECL biosensor for cancer cells was directly accomplished using the biobarcode technique to avoid cross-reaction. Moreover, magnetic beads (MBs) for aptamer immobilization were combined with a dendrimer/QD NCs probe for signal production in an ECL assay of cancer cells, which simplified the separation procedures. In particular, a cycle-amplifying technique using a DNA device on MBs was further employed in the ECL assay of cancer cells, which greatly improved sensitivity [70].

### 3.5. Chemical Substances

A large number of both organic and inorganic compounds are related to good physical condition and disease biomarkers. A group of chemical substances including drugs and ions are described in company with nanoparticles that enhance the sensitivity of the analysis in the following section.

#### 3.5.1. Electrochemical Detection

The side effects of chemical substances, namely drugs used for treating acute pain, cancer, heart conditions, bacterial infections, neulogical disorders, and respiratory disorders, are concentration dependent, and for this reason, their accurate quantification is critical. In this section, electrochemical detection using nanoparticles as modifiers are reviewed according to the sequential chemical substances listed above.

Nimesulide, zolmitriphan, acetaminophen or paracetamol (*N*-acetylaminophenol) are drugs used as analgesics. Nimesulide in medical tablets was detected by magnetic nanoparticles modified on a glassy carbon electrode. The results had a remarkable catalytic and enhancement effect on the reduction of the analyte and the reduction peak shifted positively compared with a bare electrode [10]. Silver nanoparticles/multiwalled carbon nanotube-modified glassy carbon electrodes showed an enhancement of oxidation peaks and were applied for the detection of zolmitriphan without interferences. The method was simple, sensitive, and reproducible [71]. The electrochemical detection of acetaminophen was reported in numerous references [72,73,74]. For example, electrochemically reduced graphene (ERG)-loaded nickel oxide (Ni_2_O_3_-NiO) nanoparticles coated onto a glassy carbon electrode displayed high electrocatalytic activity ascribed to the synergistic effect of the special composite structure and the physical properties of nickel oxide nanoparticles and graphene [73]. Acetaminophen had also been simultaneously detected in the presence of ascorbic acid, dopamine, and uric acid. Its detection limit as low as 0.05 µM benefited from coupling phenylethynyl ferrocene thiolate (Fc-SCA)-modified Fe_3_O_4_@Au NPs with a graphene sheet/chitosan modified glassy carbon electrode. The modified electrode exhibited synergistic catalytic and amplification effects towards the analytes [75].

Adriamycin is a trade name of doxorubicin, a drug known for cancer treatment. Its presence in calf thymus DNA was quantified with a glassy carbon electrode modified with silver nanoparticles and multiwalled carbon nanotubes with carboxy groups. The results showed excellent stability and low limit of detection [76].

Amiodarone, atenolol, and digoxin are employed in patients with heart disorder conditions, *i.e.*, cardiovascular disease and cardiac dysrhythmias. Magnetic nanoparticles were combined with a modified electrode to determine the amount of drugs. For amiodarone and atenolol, the nanoparticles were loaded in crystalline material-41 grafted with 3-amiropropyl groups and modified on a carbon paste electrode. The anodic peak currents increased owing to the adsorption of amiodarone and atenolol [77]. Magnetic nanoparticles was coated on core-shell gold nanoparticles (Fe_3_O_4_-Au-NPs), labeled with antigen, and later modified on a screen-printed carbon electrode for the analysis of digoxin with a low detection limit. The modified electrode offered simplicity, low cost, high sensitivity, stability, and reliability [78].

Anti-bacterial drugs are ubiquitous since encounters with bacteria are inevitable. Oxacillin and rifampicin are examples of anti-bacterial drugs that can be detected by electrochemistry. With the fabrication of an indium tin oxide electrode including cobalt nanoparticles, the resulting sensor had an excellent selectivity and amplification of the electrochemical response signal for oxacillin detection in human blood serum samples [79]. Nickel hydroxide nanoparticles-reduced graphene oxide nanosheets (Ni(OH)_2_-RGO) were prepared layer-by-layer on a graphene oxide (GO) film pre-cast on a glassy carbon electrode surface. The modified electrode exhibited a distinctly higher activity for the electro-oxidation of rifampicin. The peak currents was enhanced as a result of the fast electron transfer kinetics that arose from the excellent properties of RGO nanosheets and the exclusive properties of nanoparticles [80].

Chlorpromazine, clonazepam, and thioridazine are drugs used in the management of psychotic conditions. Cobalt nanoparticles modified on a carbon paste electrode showed high sensitivity for the detection of chlorpromazine in biological samples [81]. A glassy carbon electrode composed of silver nanoparticles and multiwalled carbon nanotubes was fabricated and had high electrocatalytic activity toward the reduction of clonazepam. Nanodiamond graphite, in addition, was decorated with silver nanoparticles for thioridazine detection. The enhancement in microscopic area and strong absorption of thioridazine increased the peak currents and offered high sensitivity. Moreover, the modification of nanoparticles displayed high stability, uniformity, and reproducibility [17].

Silver nanoparticles functionalized on various electrodes were used as sensitive tools for respiratory disorder drugs, for example, difficult breathing and tuberculosis. Amperometric detection of isoniazid exploiting a screen-printed carbon electrode modified with silver hexacyanoferrate in simulated human urine samples exhibited a detection limit as low as 2.6 µM [82]. Melamine functionalized silver nanoparticles were immobilized on the surface of an electrode and used for the determination of clenbuterol in biological fluids and illegal usage in livestock feeding. The approach had advantages of simplicity, rapid detection, highly sensitivity, and selectivity [83].

#### 3.5.2. Colorimetric and Spectrophotometric Detection

Chemical substances including drugs and ions can be detected by optical methods and provide high sensitivity and selectivity similar to electrochemical detection. Sample analytes in this part are divided into anti-bacterial and antiviral drug, drugs for relieving heart failure, pain, skeleton muscle performance, and ions.

Entecavir and 5-fluorocytosine are used as antiviral and antimitotic drugs, respectively. The detection concept of both analytes was based on the aggregation of modified silver nanoparticles. The change from a dispersion to an aggregation resulted in a color change from yellow to wine red in the presence of entecavir. The aggregation was due to neutralization of the electrostatic repulsion. This method was applied to real samples of human urine [84]. Along with *p-*aminobenzenesulfonic acid and silver nanoparticles, 5-fluorocytosine caused a color change from yellow to green and a SPR shift. The aggregation gaver a green color to the solution due to an electron donor/acceptor reaction. The result was detectable by the naked eye at concentrations as low as 0.08 ppm [85].

Plasmon absorbance decreased with increasing amounts of captopril, a drug for hypertension and some types of heart failure. Modified silver nanoparticles composed of ascorbic acid as a reducer and sodium dodecyl sulfate as stabilizer were used as probe. The results exhibited a low detection limit in pharmaceutical formulation samples [86].

The tripan family drugs are used for migraines and cluster headache treatment. Silver nanoparticles were capped by citrate and utilized for detection. The results were observed as a color change from yellow, to orange, and to brown. The simple, sensitive, and rapid technique was employed in pharmaceutical tablets and nasal sprays [87]. Semiconductor CdTe quantum dots were fabricated as S-β-CD-MSA-CdTe. In the presences of acetylsalicylic acid (ASA), the fluorescence was enhanced. The method could be employed for detection of aspirin [88].

An inhibition of the chemiluminescent signal was observed after the addition of baclofen into l-cysteine-capped CdS quantum dots, KMnO_4_, and Na_2_S_2_O_3_. The technique was applied for the detection of spasticity drugs showing higher sensitivity than CD-IMS [89].

Many ions act as a good representatives for disease and abnormal health status detection. For example, calcium ion as illustrative of bone condition and iodated salt as a health indicator are described [90,91]. Amino-functionalized carbon dots mixed with glutamic acid and hyaluronic acid were found to bind at bone cracks. Thus, the process was able to locate micro-cracks and map calcium deposition by fluorescence imaging [90]. Conjugated polyelectrolyte-stabilized silver nanoparticles were synthesized as light absorbers and 4-oxo-4(pyren-1-ylmethoxy) butanoic acid was used as an ideal fluorescence probe. Light absorbers quenched the ideal fluorophore and could be recovered after the addition of hydrogen peroxide and iodine ion based on the inner filter effect (IFE). The technique was used for determination of an iodate salt in urine samples [91].

### 3.6. Hormones

A hormone is a chemical messenger produced by the endocrine system that regulates body physiology and behavior such as growth and development, metabolism, sexual function, reproduction and mood. Endocrine glands secrete hormones directly into the blood, which transports the hormones through the body. Cells in a target tissue have receptor sites for specific hormones. However, too much or too little of a certain hormone can be serious. It takes only a tiny amount to cause big changes in cells or even the whole body. Thus, various laboratory tests for measuring hormone levels in biological fluids have been reported.

#### 3.6.1. Electrochemical Detection

A simple, cost effective, selective and sensitive detection method is required for routine monitoring of the endocrine-disrupting compounds in real samples. Thus, an electrochemical aptasensor for endocrine disrupting 17β-estradiol based on a poly(3,4-ethylenedioxylthiopene) (PEDOT) doped with gold nanoparticles (AuNPs) platform has been reported. The prepared electrode was employed for the immobilization of biotinylated aptamer for the detection of the target. The electrochemical signal generated from the aptamer-target molecule interaction was monitored electrochemically using square wave voltammetry in the presence of [Fe(CN)_6_]^−3/−4^ as a redox probe [92]. Moreover, an aptamer-based label free electrochemical biosensor was also used for 17β-estradiol detection. In that work, an aptamer was immobilized on a layered tungsten disulfide nanosheets/gold nanoparticle-modified glassy carbon electrode through Au-S interaction. The layered tungsten disulfide nanosheet/AuNPs film acted as an efficient platform for the assembly of bio-probes. After blocking with bovine serum albumin, the aptamer probe was then bound with the addition of 17β-estradiol to form an estradiol/aptamer complex on the electrode surface, which led to a significant decrease in peak current. The aptamer sensor holds great promise of sensitivity, reproducibility and could be extended to other analytes [93].

In addition, determination of insulin was also established by using a nickel oxide nanoparticles modified Nafion-multiwalled carbon nanotubes screen printed electrode (NiONPs/Nafion-MWCNTs/SPE). Cyclic voltammetric studies showed that the NiOPs/Nafion-MWCNTs film modified SPE lowered the overpotentials and improved the electrochemical behavior during insulin oxidation, as compared with the bare SPE. Moreover, amperometry was used to evaluate the analytical performance of the modified electrode in the quantitation of insulin. Excellent analytical performance was achieved under optimized conditions [94].

#### 3.6.2. Colorimetric and Spectrophotometric Detection

Using multifunctional gold nanoparticles (AuNPs), a surface plasmon resonance (SPR) biosensor was developed for insulin detection in human serum. Bifunctional hydroxyl/thiol-functionalized fourth-generation polyamidoamine dendrimer (G4-PAMAM)-encapsulated AuNPs were synthesized and immobilized on a gold surface. Part of the dendrimer thiol groups were converted to hydrazide functionalities providing an activated surface available to subsequently immobilize the receptor. Herein, the resulting AuNPs dendrimer-modified surface provided an assay with high stability, significantly enhanced sensitivity, and a detection limit for analyzing insulin of 0.5 pM. The SPR detection of insulin was amplified due to the changes in the dielectric properties of the matrixes, occurring upon the biorecognition processes on the sensor surface, through the coupling of the localized plasmon of the NPs with the surface plasmon wave [95].

Europium (Eu(III)) chelate-bonded silica nanoparticles have been developed as a fluorescent label for a time-resolved immunofluorometric assay (TrIFA) for human thyroid stimulating hormone (hTSH). The fluorescent nanoparticle label allowed directly reading of the fluorescent signal, omitting the signal development step required for the commercial dissociation-enhanced lanthanide fluorescence immunoassay (DELFIA) system. In combination of high sensitivity, short period of assay time, this developed method can be potentially used in hospitals for daily clinical practice in hTSH screening [96].

### 3.7. Lipids

Most of the fat found in food is in the form of triglycerides, cholesterol, and phospholipids. High cholesterol is one of the major controllable risk factors for coronary heart disease, heart attacks and strokes. Many people do not know their cholesterol is too high because there are usually no symptoms. Therefore, the monitoring of cholesterol level is very important for clinical diagnosis. Many analytical methods have been developed for cholesterol determination using either enzymatic or non-enzymatic based methods.

#### 3.7.1. Electrochemical Detection

To the present, electrochemical methods have been widely used to measure cholesterol levels. A gold nanoparticles (AuNPs)-modified cholesterol oxidase-based bioelectrode has been fabricated for the amperometric detection of cholesterol in human serum samples. The fabrication procedure was based on the deposition of AuNPs on a 1,6-hexanedithiol-modified gold electrode, functionalization of the surface of the deposited AuNPs with carboxyl groups using 11-mercaptoundecanoic acid and then covalent immobilization of cholesterol oxidase on the surface of the AuNPs film using *N*-ethyl-*N*′-(3-dimethylaminopropyl carbodimide) and *N*-hydoxysuccinimide ligand chemistry. The AuNPs provided an environment for enhanced electrocatalytic activities and thus resulted in an enhanced analytical response [97].

Besides, a porous tubular silver nanoparticles (AgNPs)-modified glassy carbon electrode (GCE) was also constructed as a working electrode for non-enzymatic cholesterol detection. The modified electrode showed markedly improved electrocatalytic activity toward cholesterol oxidation compared with solid Ag nanorods [98]. Likewise, a AgNPs-modified glassy carbon electrode (AgNPs/GCE) fabricated by an electrochemical deposition technique has also been reported for the determination of cholesterol in bovine serum based on coupling of enzymatic assay and electrochemical detection. The AgNPs catalyst possesses catalytic activity in hydrogen peroxide reduction, with no observed interference from easily oxidizable species such as ascorbic acid and uric acid. In addition, the main advantages for the use of AgNPs/GCE are in term of high sensitivity, high accuracy, and simple fabrication [99].

#### 3.7.2. Colorimetric and Spectrophotometric Detection

Several examples of elctrochemiluminescence (ECL)-based nanoparticles for cholesterol detection have been demonstrated. For example, an ECL biosensor based on an anodic ECL of luminol at low potential has been demonstrated. Firstly, C-60 was functionalized with l-cysteine (l-cys) giving an l-cys-C-60 composite, which was modified onto the surface of glassy carbon electrodes for adsorbing gold colloidal nanoparticles (AuNPs). Subsequently, cholesterol oxidase (ChO_x_) was dropped onto the surface of the modified electrode to fabricate a cholesterol biosensor. This approach promises good reproducibility, stability and anti-interference ability [100].

In a second example, an ECL biosensor for cholesterol detection based on multifunctional core-shell structured microspheres (Fe_3_O_4_@SiO_2_-Au@mpSiO_2_) has been reported. These microspheres consist of a core of silica-coated magnetite nanoparticles, an active transition layer of gold nanoparticles (AuNPs) and a mesoporous silica shell. The microspheres possess a large surface area that can increase enzyme loading and an active transition layer AuNPs can also enhance the ECL signal, providing a better analytical performance [101].

### 3.8. Microorganism

Over millions of years, microbes and humans have formed a unique relationship. The huge majority of the microbes in the body are rendered harmless by the protective effects of the immune system. Microbes are even useful for various applications such as a source of antibiotics and vaccines to treat and prevent infectious diseases, but there are many ways that bacteria and other microbes can negatively affect human life.

#### 3.8.1. Electrochemical Detection

A regenerating electrochemical impedance immunosensor has been constructed for the detection of type 5 adenovirus. The multi-layered immunosensor fabrication involved modification steps on gold electrodes: (1) modification with a self-assembled monolayer (SAM) of 1,6-hexanedithiol to which gold nanoparticles (AuNPs) were attached via the distal thiol groups; (2) formation of SAM of 11-mercaptoundecanoic acid onto the AuNPs; (3) covalent immobilization of monoclonal anti-adenovirus 5 antibody, with EDC/NHS coupling reaction on the nanoparticles. The immunosensor displayed a very good detection limit of 30 virus particles/mL and a wide linear dynamic range. An electrochemical reductive desorption technique was employed to completely desorb the components of the immunosensor surface, then re-assemble the sensing layer and reuse the sensor [102].

#### 3.8.2. Colorimetric and Spectrophotometric Detection

Recently, a platform based on conjugating long spacer arms (LSA) of carboxymethylated glucan (CMG) onto magnetic nanoparticles (MNPs) was developed to enhance the chemiluminescence (CL) detection of infectious pathogens (hepatitis B virus (HBV)). CMG-MNPs are designed to have low steric hindrance and high suspension properties, which allow for facile modification and hybridization reactions that enhance the CL sensitivity and detection. The biotinylated amplicon of HBV was hybridized to DNA probes functionalized on CMG-MNPs. The magnetic complexes were then incubated with streptavidin-alkaline phosphatase (SA-AP) to form linkages. Finally, the magnetic complexes were mixed with AMPPD to generate a CL signal that is proportional to the concentration of HBV target. When optimized, the platform showed high specificity and a detection limit of 0.5 pM, which exhibited great promise for the early clinical diagnosis of infectious diseases [103]. In addition, a method for the detection of RNA virus (hepatitis C virus (HCV)) based on enzyme free MNPs extraction of nucleic acid and chemiluminescence has been reported. The specific lysis buffer conditions helped the nucleic acid adsorb on the surface of MNPs. The CL detection of HCV was achieved by incubating the biotin labeled RT-PCR products with probe-labeled MNPs and streptavidin-alkaline phosphatase (SA-AP) [104].

**Figure 3:**
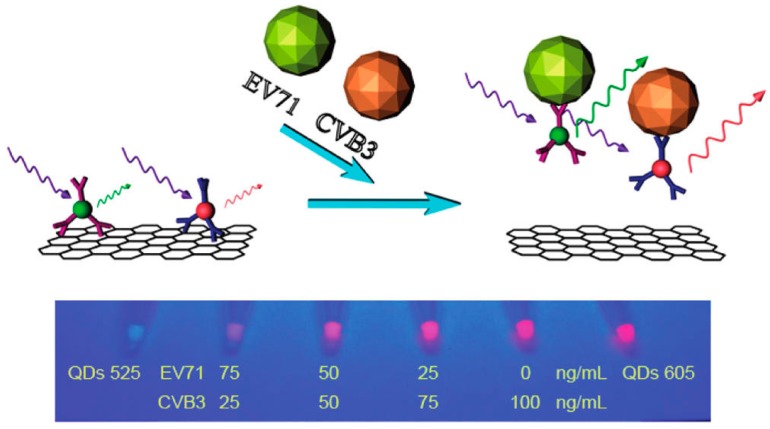
Schematic Presentation of the Multicolored QDs-Ab and GO Based EV71 and CVB3 Determination Biosensor and photovisualization of semiquatitative simultaneous determination of EV71 and CVB3 [105]. Reprinted with permission from (Chen, L.; Zhang, X.; Zhou, G.; Xiang, X.; Ji, X.; Zheng, Z.; He, Z.; Wang, H. Simultaneous determination of human enterovirus 71 and coxsackievirus b3 by dual-color quantum dots and homogeneous immunoassay. *Anal. Chem.* **2012**, *84*, 3200–3207.). Copyright (2012) American Chemical Society.

Simultaneous determination of human enterovirus 71 (EV71) and coxsackievirus B3 (CVB3) was also possible using dual-color quantum dots (QDs), as shown in Figure 3. The QDs are streptavidin-conjugated quantum dots (SA-QDs), and the antibodies are biotinylated antibodies. Biotinylated EV71 antibody (Ab_1_) was associated with 525 nm green colored SA-QDs via biotinstreptavidin interaction forming QDs-Ab_1_, whereas biotinylated CVB3 antibody (Ab_2_) was associated with 605 nm red colored SA-QDs via biotinstreptavidin interaction forming QDs-Ab_2_. Graphene oxide (GO) was a quencher to the flurescent of both QDs-Ab_1_ and QDs-Ab_2_. The target of EV1 and CVB3 can break up the complex of QDs-AB and GO, recovering the fluorescence of QDs-Ab_1_ and QDs-Ab_2_, respectively. Using these dual-color QDs, the two enterovirus can be simultaneously quantitatively determined with a single excitation light [105].

### 3.9. Neurotransmitters

Neurotrabnsmitters have received considerable attention due to their important role in the human brain and body [106]. Dopamine has been recently an interesting target analyte of many research groups. Its deficiency causes cognitive malfunctions, such as Parkinson’s disease [107]. Other neurotransmitters, *i.e.*, l-dopa, choline, and serotonin, also have essential role in animals.

#### 3.9.1. Electrochemical Detection

Gold nanoparticle-modified electrodes have been individually and simultaneously used for detection of dopamine and l-dopa. For example, a gold nanoparticles functionalized carbon nanotubes (AuNP-CNT)-modified pyrolytic graphite electrode (AuNP-CNT/PGE) decreased the oxidation potential whereas oxidation currents were increased by five-fold compared with PGE in the determination of l-dopa [107]. Dopamine was detected simultaneously in urine serum with a modified glassy carbon electrode. The biosensor was fabricated by electrodeposition of Au-nanoclusters on a poly(3-amino-5-mercapto-1,2,4-triazole) (p-TA) film-modified glassy carbon electrode (GCE). The combination of materials produced a large surface area electrode and provided biological compatibility as well as good conductivity, stability, high sensitivity, and selectivity [108].

Ascorbic acid was a serious interference in the analysis of dopamine, thus a negatively charged electrode surface using PEGylated arginine functionalized magnetic nanoparticles was fabricated to resolve the problem. Dopamine interacted with the charged electrode and exhibited high sensitivity and selectivity for detection at a low concentration [109]. The fabrication of glassy carbon electrode modified-NiF_2_O_4_ magnetic nanoparticles decorated with multiwalled carbon nanotubes showed a synergistic effect toward the oxidation of dopamine without interference from other organic compounds, especially ascorbic acid, uric acid, cysteine, and urea. The results indicated a low detection limit and wide linear dynamic range for dopamine quantification in pharmaceutical, urine, and human blood serum samples [110].

A nanocomposite film of choline oxidase, multiwalled carbon nanotubes, gold nanoparticles, and poly(diallyldimethylammonium chloride) (PDDA) was used for choline detection. Gold nanoparticles was coated on the multiwalled carbon nanotubes by the interaction of gold and thiols. PDDA was utilized as dispersant and binder material. The film had good reproducibility and long term stability with anti-interference ability [111]. Other nanocomposites of cuprous oxide nanoparticles [112], gold nanoparticles [113,114], palladium nanoparticles [106], and zinc oxide nanosheets [115] present corresponding advantages resulting from their nanoparticles, including excellent sensitivity, selectivity, coupled with good stability.

Dopamine in commercial available human serum samples was analyzed without interferences using chitosan-stabilized silver nanoparticles and *p*-toluenesulfonic acid-doped ultrathin polypyrrole film. The results indicated a low detection limit of 0.58 nM [116]. Dopamine was detected simultaneously in the presence of uric acid and ascorbic acid [117,118]. For example, a silver nanoparticles-decorated reduced graphene oxide composite (AgNPs/rGO) was responsible for excellent electrocatalytic activity and well separated oxidation peaks. The method had good stability, sensitivity, and selectivity. It was applied for detection of these three compounds in commercial pharmaceutical samples, such as vitamin C tablets and dopamine injections [117]. Other neurotransmitters, namely serotonin, were detectable in plasma serum samples with a detection limit as low as 0.15 mu·M by utilizing a platinum electrode modified with multiwalled carbon nanotubes, polypyrrole, and colloidal silver nanoparticles [19].

Other metal nanoparticles were coated onto electrodes for the determination of dopamine, epinephrine, and l-dopa. To illustrate this, palladium nanoparticles with multiwalled carbon nanotubes and ionic liquids were decorated onto a carbon paste electrode for the simultaneous detection of dopamine, ascorbic acid, and uric acid. The results displayed three sharp and well separated peaks. The detection limit was in the nM range [119]. Epinephrine was detected in the presence of dopamine by square wave voltammetry. A glassy carbon electrode modified with nickel oxide nanoparticles and carbon nanotubes within a dihexadecylphosphate film was used for analysis of these compounds in human body fluids consisting of cerebrospinal fluid, human serum, and lung fluid [120]. Cobalt hydroxide nanoparticles and multiwalled carbon nanotubes was constructed on a carbon ionic liquid electrode for the detection of l-dopa and melatonin in pharmaceutical and human urine samples. The electrode provided high sensitivity as well as convenient preparation and high stability [121].

#### 3.9.2. Colorimetric and Spectrophotometric Detection

Recent applications show that modified gold nanoparticles can be used for the visual detection of dopamine. For example, the use of BSA-stabilized Au nanoclusters (BSA-AuNCs) showed dramatically decreased the fluorescence intensity that could be inhibited upon the addition of dopamine. The signal decrease was due to the dopamine electrostatically attached to the BSA-AuNCs [122]. Moreover, melamine-induced aggregation of gold nanoparticles was inhibited in the presence of dopamine. The color changed from red to blue and could be detected by the naked eye. The aggregation was a consequence of strong hydrogen bonding between melamine and dopamine [123]. Dopamine was capped on the surface of gold nanoparticles and subsequently caused the aggregation. The reaction was catalyzed by thioglycolic acid that was modified through hydrolysis promoting Au-S bond formation. The addition of dopamine changed the solution color from red to purple, or red to yellow. The method provided a detection limit lower than the level of existence of dopamine in urine [124].

Many magnetic nanoparticles are reported to have peroxidase mimetic activity. As an illustration, Co_x_Fe_3−x_O_4_ magnetic particles were synthesized and could effectively catalyze the reaction between 3,3,5,5-tetramethylbenzedine or TMB and H_2_O_2_. Dopamine, which possesses a hydroxyl group, was able to react as a reducing agent and consume the H_2_O_2_. The blue color due to the interaction between TMB and H_2_O_2_ is eventually faded. The technique could quantify dopamine in human serum samples at low µM levels [125].

Silver nanoparticles have the ability to enhance the co-luminescent effects of rare earth ions such as Tb^3+^ and Y^3+^. Dopamine could increase the luminescent intensity of the ions. The analyte in hydrochloride injection was determined at concentrations as low as nM level using this metal-enhanced fluorescence concept [126].

### 3.10. Nucleic Acids

Nucleic acids are nucleotide biopolymers. Nucleotides are composed of 5-carbon sugars, phosphate groups, and nitrogenous bases. RNA and DNA are types of nucleic acid. The two have different sugars, thus RNA is composed of ribose, whereas DNA is composed of deoxyribose. The nitrogenous bases are derivatives of pyrimidine (*i.e.*, cytosine, thymine, and uracil) and purine bases (*i.e.*, adenine and guanine). DNA and RNA are associated with gene expression. NADH is two nucleotides joined together at a phosphate group or so-called dinucleotide. It is a coenzyme that carries electrons from one reaction to another. Due to the prevalence of these substances in living cells, the detection of nucleic acids and their composition is important. 

#### 3.10.1. Electrochemical Detection

Gold nanoparticles were modified onto a mercapto-diazoaminobenzene monolayer-modified electrode (AuNPs-ATP-diazo-ATP) based on self-assembly for the detection of complementary single-stranded DNA. The DNA was determined with a detection limit of 9.10 × 10^−11^ M by differential pulse voltammetry with the use of Co(phen)_3_^3+^ as an electrochemical indicator. The gold nanoparticles were assumed to be responsible for the efficient electron transfer ability. The modified electrode had in consequence good selectivity and was easily regenerated [127].

Magnetic nanoparticles were immobilized with linker DNA and CdS NPs for the hybridization of the target DNA. Utilizing the nicking endonuclease for cutting the specific strand of linker DNA, the target DNA was liberated and able to re-hybridize with other modified nanoparticles. When the linker DNA was transected, the CdS NPs was released. Hence, the amount of released nanoparticles was enhanced. The amplification signal was detected by SWV with a detection limit of 0.08 fM. The method possessed high sensitivity, satisfactory reproducibility, and excellent stability [128]. Fe_3_O_4_ magnetic nanoparticles were loaded onto the surface of a MWC-NTs-modified GC electrode for the detection of NADH by amperometric detection. The modified electrode presented the advantages of both MNPs and MWC-NTs. Fe_3_O_4_ has similar redox properties to mediators that favor the electron transfer between NADH and the electrode. As a result, the modified electrode catalyzed the oxidation of NADH at low potential and the overpotential was decreased. NADH could be detected with this technique with a detection limit of 0.3 µM. In addition, the method was used for detection of lactate by coupled dehydrogenase enzymes with a modified electrode. The detection limit for lactate using DPV was 0.5 µM. The method promised to offer an efficient transducer for the design of biosensors [129].

A TiO_2_-graphene nanocomposite was modified on a glassy carbon electrode for the determination of purine bases, namely adenine and guanine. The detection limits of adenine and guanine were 0.10 and 0.15 µM, respectively. The electrocatalytic activity was improved because the modified electrode had a high adsorptivity and conductivity [130]. AuNPs/rGO was formed on GCE with redox mediators and enzymes for NADH detection. The modified electrode produced an excellent direct electrocatalytic oxidation of NADH due to a large active surface area and a favorable environment for electron transfer between NADH and the electrode. The electrocatalytic current density was 2–3 times higher compared with AuNPs alone. The detection limit was 1.13 nM (S/N = 3). The interferences, *i.e.*, glutathione, glucose, ascorbic acid, guanine, were negligible. The method was applied in human urine samples [131]. CdSe QDs-GO was immobilized on a paraffin wax-impregnated graphite electrode (PIGE) for adenine and guanine detection. The modified electrode showed excellent electrocatalytic activity for the oxidative determination of adenine and guanine with a good peak separation of 0.31 V. The detection limits of adenine and guanine were 0.028 and 0.055 µM, respectively. The method was employed for herring sperm DNA as an example of a real sample [132].

Poly(styrene-co-acrylic acid) microbeads were functionalized with CdTe quantum dots for the detection of DNA. The engagement of quantum dots made the polybeads an effective platform for labelling DNA and protein. The CdTe-tagged polybeads with a DNA probe specific to breast cancer was used for determination of DNA using SWV to measure Cd^2+^ after dissolution of CdTe tags with HNO_3_. The detection limit of this technique was 0.52 fM [133].

Graphene was easily coated with polydopamine and funtionalized with AgNPs in order to assemble an electrode for the detection of the nucleic acid derivatives. The confirmation of electrode doping was illustrated by comparing pre-coating images obtained by SEM, TEM, and XRD with post-coating images. HS-SSDNA was immobilized and methylene blue was utilized as an electrochemical indicator for the determination of DNA. The results showed the detection limit of 3.2 × 10^−15^ M (S/N = 3) and high selectivity of differentiation of one-base mismatched DNA [134]. A similar technique for the determination of adenine and guanine was reported having a detection limit of 2.0 and 4.0 nM, respectively. The modified electrode showed more favorable electron transfer kinetics than both Gr-modified GCEs and AgNPs-modified GCEs [135].

Nickel and nickel oxide nanoparticles was modified on a GCE for the detection of DNA and NADH. NADH was detected with a NiO_x_NPs/GC electrode by chronoamperometry without using any electron mediator. The detection limit was 106 nM (S/N = 3). The modified electrode possessed excellent electrocatalytic activity toward oxidation of NADH at a reduced voltage [136]. Likewise, a GCE was modified with NiNPs dispersed on PDAN for the detection of NADH. The modified electrode provided a positively synergistic effect in the electrochemical oxidation of NADH with excellent selectivity. Three types of voltammetry were employed including CV, SWV, and DPV. Each had a different detection limit of 0.378, 0.122, and 0.02 µM, respectively [137]. In another reference the immobilization of a DNA probe and a [Ru(NH_3_)_5_Cl]PF_6_ complex onto a NiO_x_(NP) modified GCE was reported. NiO_x_(NP) provided strong affinity for phosphate groups, thus oligonucleotide probes with a terminal phosphate group can be attached to the surface of the modified electrode for the detection of DNA. The detection limit was 6.8 × 10^−11^ M. The Ru-complex only responded to the complementary sequence of DNA. The technique has the advantages of good selectivity, good sensitivity, excellent reproducibility, stability, and simplicity [138].

#### 3.10.2. Colorimetric and Spectrophotometric Detection

The Stx-2 gene that causes disease in human was used as an example of colorimetric detection by gold nanoparticles (GNPs). GNPs were functionalized with thiolated ssDNA complementary to a target specific based-pair of stx-2 genes [139]. A detection and capture probe were reported in another reference for miRNA determination. The detection probe consisted of thiol-DNA and gold nanoparticles, while the capture probe had biotin-single strand DNA as a combination. Adivin immobilized on a flow strip could capture the adivin-biotin-Au-sample complex so that miRNA was detected at levels as low as 1 fM without silver enhancement [140].

Magnetic nanoparticles were functionalized for the detection of DNA. There were two probes for trapping and sensing, respectively. Complementary DNA was covalently immobilization on the target for the fabrication of the trapping probe, which had the responsibility of concentrating the target DNA from complex. The detection probe, on the other hand, was made from Fe_3_O_4_@Al_2_O_3_ magnetic nanoparticles and riboflavin-5-monophospahte (RFMP) through Al-phosphate chelation. RFMV is a fluorescent dye used in a reaction in which the displacement of DNA on the probe would release RFMP into the solution and enhanced fluorescent intensity. Ultimately, the DNA remaining on the probe was detected by MALDI-MS [141]. Target mutant DNA with a hairpin loop portion consisted of biotin at 3′ and isothiocyanate (FITC) at 5′ forming a nicking site for nicking endonuclease (NEase). NEase would cleave the hairpin and DNA, releasing parts with fluorescence for signal detection and DNA that was able to initiate the recycling process. Hence, the amount of fluorescence was increased and could be quantified. The method was applied for the detection of p53 gene and had high selectivity toward mismatched DNA [142].

HIV-1 and HIV-2 at a single molecule level could be detected by functionalized quantum dots (QDs). The QDs had two functions. One was to act as a concentrator, and another as fluorescence pair. The technique was simple, showing high sensitivity and low sample consumption with short analysis times [61].

The determined of adenine with magnetic nanoparticles decorated with silver nanoparticles via photochemical reduction was proposed. The modified particles had a seven-fold signal enhancement and could be used for the detection of adenine at a few hundred nanomolar concentration [143].

A sandwich-type DNA sensor was fabricated. Magnetic nanoparticles were functionalized at the amino group of DNA, whereas the other DNA had a carboxyl functionalized by gold nanoparticles. Cobalt nanoparticles were modified onto DNA-functionalized gold nanoparticles to amplify the detection signals. Later, the mixture of functionalized magnetic and modified gold nanoparticles would liberate cobalt ions in the presence of acid, and the chemiluminescence of the cobalt ions could be detected in the presence of luminol and H_2_O_2_ with a low detection limit [144].

### 3.11. Proteins

Proteins are large biomolecules composed of amino acids that polymerizes to polypeptides. Proteins are essential compounds to many living beings, especially animals. They influence health through dietary consumption, and protein deficiency affects growth, therefore, the quantification of proteins is important. There are many forms of protein that play critical roles in the living systems, but most publications refer to thrombin due to its crucial property as a part of the blood coagulation system. Other protein and protein derivative analytes are also described in the following subsections.

#### 3.11.1. Electrochemical Detection

Gold nanoparticles with antithrombin-aptamer as molecular recognition element improved the sensitivity for thrombin detection. The combination was self-assembled on the surface of a bare electrode using 1,6-hexanedithiol. The redox couple was monitored for the electron transfer resistance of the aptasensor. The detection limit was 0.013 nM. The aptasensor showed good selectivity toward thrombin against other proteins [145]. Another approach presented an aptamer-gold nanoparticles-horseradish peroxidase (HPR) sensor for the detection of thrombin. A capture probe of aptamer 1 immobilized onto the core/shell Fe_3_O_4_/Au magnetic nanoparticles and a detection probe of aptamer 2 labeled with AuNPs and HRP was fabricated. Thrombin linked the captured and detection probe together causing an amplification of the signal. The method was simple, rapid, and selective. It showed great performance in the determination of thrombin in disease diagnosis [146].

**Figure 4:**
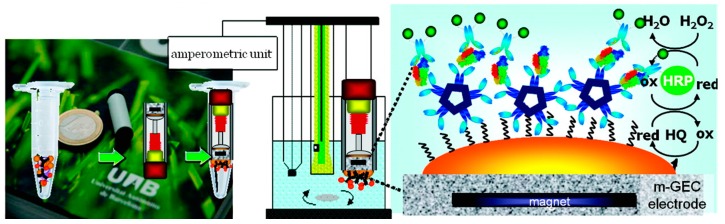
Schematic representation of the experimental details of the P. falciparum antigen (HRP2) related to malaria disease in human serum for the electrochemical magneto immunosensor [148]. Adapted with permission from (de Souza Castilho, M.; Laube, T.; Yamanaka, H.; Alegret, S.; Pividori, M.I. Magneto immunoassays for plasmodium falciparum histidine-rich protein 2 related to malaria based on magnetic nanoparticles. *Anal. Chem.* **2011**, *83*, 5570–5577.). Copyright (2011) American Chemical Society.

An electrochemical platform based on Fe_3_O_4_@polyaniline nanoparticles (Fe_3_O_4_@PANI NPs) has been developed for the detection of creatinine in human plasma and urine. The creatinine target molecule was self-assembled on the surface of nanoparticles through N-H hydrogen bonding. Molecular imprinted polymers (MIPs) were established on a magnetic glassy carbon electrode. The sensors exhibited high sensitivity and selectivity for creatinine with a low detection limit [147]. The electrochemical signals modified by micro- and nanoparticles for detection of *Plasmodium falciparum* histidine-rich protein (HPR2) that is related to malaria were compared. The particles were immobilized with HPR2-antibody and horseradish peroxidase (HRP), and then captured by graphite-epoxy composite (m-GEC) which was used as the transducer for the detection. The results showed that magnetic nanoparticles had better analytical performance for a rapid, simple, cost-effective, and on-site detection of HRP2 in blood [148]. The schematic of the detection method is illustrated in Figure 4.

Graphene/3,4,9,10-perylenetetracarboxylic acid (GPD) with a three-dimensional porous structure had been fabricated as a redox probe. The novel probe has a high electrochemically active area and conductivity. In thrombin detection, the probe showed higher sensitivity compared with other redox probes [149]. ST6Gal-I is a protein marker of tumors and cancer. It was detected using a modification of nanocomposites and gold nanoparticles. The nanocomposites were Prussian Blue-based (PB). The technique had excellent sensitivity and selectivity and could be used for quantification of the protein in human serum samples with a detection limit as low as 3 pg·mL^−1^ [150].

Silver nanoparticles decorated with ZnO nanotubes were used as tools for the detection of D-dimer. D-dimer is present in humans under thrombosis (DVT) disorder conditions. Firstly, ZnO nanorods were etched into nanotubes that later would be covered with silver nanoparticles. The biosensor exhibited a low detection limit with acceptable selectivity and reproducibility. The method promised specific detection of D-dimer in clinical and real samples [151].

#### 3.11.2. Colorimetric and Spectrophotometric Detection

Thrombin is detected by various colorimetric techniques. For example, gold nanoparticles with the addition of thrombin in an excess of fibrinogen induced the formation of insoluble fibrin-AuNPs as a result of the polymerization of the unconjugated and conjugated fibrinogen. The absorbance of the supernatants decreased as the amount of thrombin increased. This probe exhibited high sensitivity and selectivity over other proteins. The limit of detection is lower than those obtained using other nanomaterial- and aptamer-based detection methods. The technique has potential for the detection of thrombin in disease diagnosis [152]. Another reference [153] reported a technique similar to the previous one for quantification of thrombin without interferences from proteins such as bovine serum albumin, pepsin, trypsin, *etc*. The catalytic activity of gold nanoparticles in the luminol H_2_O_2_ chemiluminescent method (CL) was used for the detection of thrombin. The effective binding of the target protein and aptamer could induce the aggregation of gold nanoparticles, and subsequently enhance the CL reaction. This biosensor provided a simple, cheap, rapid, and sensitive method for detection of thrombin [154].

The parallel concept of aptamer-induced aggregation was applied in magnetic nanoparticles for thrombin detection. Gold-coated iron oxide nanoparticles were synthesized. The synthesized nanoparticles had a flowerlike or nanorose shape. The nanorose changed from well dispersed to an aggregated state in the presence of human-α thrombin, which resulted in an alteration of the UV-Vis absorption spectra. The dual detection of qualification and quantification was advantageous as it provided more reliable results. The technique was used for detection of thrombin at low detection limits [155]. Likewise, magnetic nanoparticles with antithrombin aptamer conjugated on SPR gold film was used for detection of thrombin by SPR spectroscopy with a low detection limit. The selectivity of the technique was tested using three kinds of protein including BSA, human IgM, and human IgE. The results showed that the nanoparticles were an excellent amplification reagent in SPR detection of thrombin and had great performance [11]. Other approaches were employed for the quantification of thrombin, the signal enhancement being due to core-shell gold capped magnetic nanoparticles (GMPs) was an example. In a solution comprised of thrombin aptamer 1 and GMP5-Apt2 conjugates a remarkably increase in SPR angle was displayed due to the larger mass and higher refractive index of GMP5-Apt2 compared with gold nanoparticles. Thus, a low level detection limit could be achieved. The method presented a novel option for protein detection and disease diagnosis [156]. Besides thrombin, various analytes such as C-reactive protein (CRP), bovine serum albumin (BSA), and cardiac troponin I (cTnI) have utilized magnetic nanoparticles as a significant features for colorimetric detection [4,7,157].

Quantum dot coupling with thrombin aptamer was used for the quantification of thrombin. QDs-apt:B was constructed by assembling the quantum dots with a single-stranded aptamer, then staining the duplex regions of the aptamer with a DNA intercalating dye (BOBO-3). The dye was released as thrombin induced the folding of the aptamer. The QD fluorescence resonance energy transfer (FRET)-mediated BOBO-3 emission decreased, therefore, the technique could be applied to evaluate the thrombin level [158]. Moreover, quantum dots functionalized with aptamer as well as magnetic nanoparticles functionalized by aptamer showed ability for the determination of thrombin with low detection limits. The system was assembled on a chip using fluorescence as a detection technique. The results revealed that the on-chip platform had advantages of speed and efficiency [159].

A protein derivative such as an amino acid can be detected using nanoparticles too. For example, short peptide chains were hydrolyzed into the negative peptide apart and positively charged dipeptides, in the presence of trypsin. The dipeptides were able to cap on the surface of silver nanoparticles and induce aggregation under salt conditions. The interaction caused a change in the color of the solution, which could be detected by UV-Vis spectrophotometry and the naked eye. This technique provided a novel strategy for trypsin determination in clinical applications [160]. An aptamer was functionalized on silver nanoparticles (apt-AgNPs) for the determination of platelet derived growth factor-BB (PDGF-BB) protein. First, the aptamer and ssDNA were loaded on the nanoparticle surface to form a probe. Second, the probe could cause metal deposition by catalyzing the reduction of metallic ions in a color agent. The corresponding results could be captured by the naked eye. There were two coloring agents, namely silver enhance solution, and color agent 1 which was a solution of HAuCl_4_ and hydroquinone. The results demonstrated that color agent 1 had benefits over the other option, especially at low detection limits due to its superior sensitivity. The development showed good potential in complex biological samples [29].

### 3.12. Sugars

Diabetes is a metabolic disease, which can cause serious health effect such as heart disease and kidney failure. Thus, monitoring of glucose levels is required for the diagnosis and control of diabetes. For this purpose, various analytical methods based on the use of the nanomaterials have been established for glucose monitoring.

#### 3.12.1. Electrochemical Detection

According to the sugar determination, this approach can be classified into two main groups: enzymatic and non-enzymatic methods. Enzymatic methods are highly selective, fast and sensitive, but several parameters can affect enzyme activity. To overcome these disadvantages, many glucose sensors that have been made are based on non-enzymatic methods such as those of Ismail *et al.* [161] who reported a non-enzymatic electrochemical glucose sensor based on a graphene oxide nanoribbon (GONRs)-gold nanoparticle (AuNPs) hybrid. They found that AuNPs supported by GONRs were greatly superior to the unsupported conventional bare gold electrode, with a greatly enhanced current density (approximately by 200%). This is attributed not only to the high total surface area of the AuNPs compared to that of a Au sheet, but also to the three-dimensional specific interaction between the functional groups on the GONRs and the Au active sites with the reactant and the intermediates that promote the reaction kinetics. Another non-enzymatic glucose sensor was also developed based on the electrocatalytic oxidation activity of nanoporous gold (NPG) toward glucose. The NPG electrode was prepared by a rapid one-step square-wave oxidation reduction cycle (SWORC). The prepared NPG electrode had high roughness, and excellent electrocatalytic activity toward glucose electrooxidation. In addition, Nafion was selected as a protective film to enhance the specificity of the developed glucose sensor with no interference from ascorbic acid and uric acid [162].

To increase the sensitivity of enzymatic glucose biosensors, several articles have been published based on the use of magnetic nanoparticles (MNPs) as an immobilization platform for glucose oxidase (GOD). A conductive-catalyst system that consisted of Fe_3_O_4_ MNPs and oxidative enzymes co-entrapped in the pores of mesoporous carbon, forming a magnetic mesoporous carbon (MMC) has been fabricated. GOD is subsequently immobilized in the remaining pores of the MMC using glutaraldehyde cross-linking to prevent enzyme leaching. H_2_O_2_ is generated by the catalytic action of GOD in proportion to the amount of the glucose and is subsequently reduced in H_2_O by the peroxidase mimetic activity of MNPs generating a cathodic current, which can be detected through the conductive carbon matrix [163]. Furthermore, a practical glucose biosensor with immobilization of glucose oxidase (GOD) enzyme on the surface of citric acid (CA)-assisted cobalt ferrite (CF) magnetic nanoparticles (GOD/CA-CF/GCE) has been reported. This approach worked on the principle of detection of H_2_O_2_ which is produced by the enzymatic oxidation of glucose to gluconic acid. This sensor has tremendous potential for application in glucose biosensing due to its higher sensitivity and a substantial increment of the anodic peak current [164]. In order to improve the sensitivity, an amperometric glucose sensor based on an enhanced catalytic reduction of oxygen using GOD adsorbed onto core-shell Fe_3_O_4_@silica@Au MNPs has been reported. The authors found that the immobilized GOD retained its bioactivity with protein load and exhibited a fast heterogeneous electron transfer rate [165]. Another work demonstrated magnetic single-enzyme nanoparticles (MSENs) encapsulated within a composite Fe_3_O_4_/poly(pyrrole-N-propylsulfonic acid) network. The MSENs were then modified onto a magnetic glassy carbon electrode (MGCE) through magnetic immobilization. The modified electrode exhibited high selectivity, stability and rapid operation for the detection of glucose [9].

Nanocomposites have also found widespread use for monitoring glucose. This is due to their greatly enhanced electrocatalytic activity toward glucose in the electrochemical detection step. In these systems, chronoampermetry or amperometry are often performed, which are very suitable for the determination of glucose. Using a PtPd/MCV nanocomposite (PtPd bimetallic alloy nanoparticles on onion-like mesoporous carbon vesicle (MCV))-modified glassy carbon electrode, a nonenzymatic glucose sensor has been reported. Compared with Pt/MCV nanocomposite, the PtPD/MCV nanocomposite displays an enhanced current response toward glucose. The particular lamellar structure of the MCV also resulted in favorable transport passage for glucose [166]. Glucose was also measured based on integration of glucose oxidase (GOD) with a Pt nanoparticles/ordered mesoporous carbon (OMC) nanocomposite-modified electrode. In this approach, GOD was immobilized by entrapment in an electropolymerized pyrrole film for the construction of a glucose biosensor, providing great sensitivity [167]. Moreover, a non-enzymatic glucose sensor based on a glassy carbon electrode has been recently modified with copper oxide (CuO) nanocubes-graphene nanocomposite [168], nickel hydroxide/graphene nanocomposite [169]. GOD immobilized on poly(methylene blue) doped silica nanocomposites (PMB@SiO_2_) was also constructed on a glassy carbon electrode. Compared with poly(methylene blue) film, PMB@SiO_2_ had more advantages in facilitating electron transfer between GOD and the electrode surface [170]. Recently, a high performance non-enzymatic glucose sensor based on polyvinylpyrrolidone (PVP)-stabilized graphene nanosheets (GNs)-chitosan (CS) nanocomposite was demonstrated. Benefitting from the synergistic effect of GNs (large surface area and high conductivity), NiNPs (high electrocatalytic activity in glucose oxidation) and CS (good film-forming and antifouling ability), this enzyme-free sensor was established with outstanding detection limits and attractive selectivity [171]. An *in-situ* polypyrrole cross-linked chitosan/glucose oxidase/gold bionanocomposite film was fabricated to constructed a simple glucose sensor. The resulting bionanocomposite provided a suitable environment for the enzyme to retain its bioactivity under quite extreme conditions, and the decorated AuNPs in the bionanocomposite offer good enzyme affinity [172]. Glucose was also measured by an enzyme-free sensor based on chemical oxidative polymerization of pyrrole monomers on the surface of CuFe_2_O_4_ nanoparticles (core-shell-CuFe_2_O_4_/PPY nanocomposite). It was shown that the presence of pyrrole increased the electronic interaction between NPs and the polypyrrole matrices [173]. To improve the sensitivity and prevent GOD from leaching away, a sensitive glucose sensor based on the immobilization of GOD on hollow Pt nanospheres assembled on graphene oxide (GO)-Prussian Blue (PB)-3,4,9,10-perylenetetracarboxylic dianhydride derivative (PTC-NH_2_) nanocomposite film has been reported [174]. Interestingly, to pursue high performance for non-enzymatic glucose sensors, the modified electrode coupling with concentrated hydroxide electrolyte based on a fast conversion of the redox couple (Ni(OH)_2_↔NiOOH: Ni^II^/Ni^III^) on Ni(OH)_2_ nanoparticles-modified carbon (Ni(OH)_2_/C) nanocomposites has been established. The excellent performance for glucose detection is attributed to the fast conversion of Ni^II^/Ni^III^ which is accelerated by an adequate hydroxide electrolyte concentration, fast electron transfer in the carbon skeleton, and high activity of Ni(OH)_2_/C nanocomposite [175].

Silver nanoparticles (AgNPs) are of interest for the electrochemical detection of glucose. Several papers have been published based on the use of AgNPs for the modification of the electrode. For example, an enzymatic glucose sensor based on immobilizing glucose oxidase (GOD) on a AgNPs-decorated multiwalled carbon nanotube (AgNP-MWNT) modified glass carbon electrode (GCE) has been reported. The AgNP-MWNT composite membrane showed improved biocompatibility for GOD immobilization and an enhanced electrocatalytic activity toward reduction of oxygen due to the decoration of AgNPs on MWNT surfaces. The AgNPs also accelerated the direct electron transfer between the redox-active site of GOD and the GCE surface because of their excellent conductivity and large capacity for protein loading, leading to direct electrochemistry of GOD [176]. In a second example, an amperometric glucose sensor based on silver nanowires (AgNWs) and chitosan (CS)-glucose oxidase (GOD) film was demonstrated. The results indicated that AgNWs play an important role in the enhanced electron transfer between the immobilized GOD and the surface of electrode, which are attributed to large surface-to-volume ratio and high conductivity of AgNWs [177]. Recently, a simple method for the decoration of graphene oxide (GO) with AgNPs as the catalyst material was used for the glucose sensor applications. The total response of the sensor was improved significantly, mainly because of the synergistic interaction between the AgNPs and GO [178].

Owing to the unique properties of the mentioned metal nanoparticles, other metal nanoparticles have also been used in non-enzymatic sensors to enhance the sensitivity. Cobalt nanoparticles (CoNPs) are alternative metal nanoparticles that have good potential for glucose sensing due to a prominent electrocatalytic activity toward glucose. For instance, a non-enzymatic amperometric sensor based on a cobalt oxide nanoparticle-modified glassy carbon (CONM/GC) electrode was reported. The modified electrode exhibited excellent performance for glucose determination, sensitivity and fast response times [179]. Moreover, a selective sensor based on cobalt oxide nanoparticles electrodeposited on reduced graphene oxide was also used, which displayed a remarkably selectivity toward glucose [180]. Another sensor has been reported using a new type of cobalt nanoparticle modified indium tin oxide electrode made by an ion implantation technique [181].

Nickel nanoparticles (NiNPs) are another type of metal nanoparticles that exhibit excellent electrocatalytic ability toward glucose broadly used in glucose sensors. Nie *et al.* [182] reported a non-enzymatic glucose sensor based on using well-distributed NiNPs on straight multi-walled carbon nanotubes (SMWNTs) nanohybrids, which were synthesized through an *in situ* precipitation procedure. The observed remarkable enhancement in electrocatalytic activity can be attributed to the synergistic effect of SMWNTs and Ni^2+^/Ni^3+^ redox couple. Nickel hexacyanoferrate nanoparticles (NiHCF)-modified TiO_2_ nanotube arrays (TNTs) were also applied for the non-enzymatic detection of glucose. NiNPs were deposited on TNTs by a pulse electrodeposition method and then converted to NiHCF by cyclic voltammetry in a solution containing [Fe(CN)_6_]^3−^ [183]. Furthermore, NiNPs electrodeposited on reduced graphene oxide film have also been reported [184]. In order to achieve a higher sensitivity, a single layer of nickel hydroxide nanoparticles (Ni(OH)_2_NPs) covered on a porous Ni foam electrode was successfully applied for the quantification of glucose by an amperometric method [185].

Palladium nanoparticles (PdNPs) are also of interest as a great potential catalyst for improving the performance of glucose sensors. For example, PdNPs modified on functional carbon nanotubes (FCNTs) have been reported. Based on the electrochemical results, PdNPs efficiently catalyzed the oxidation of glucose and showed excellent resistance towards poisoning from interfering species such as ascorbic acid and uric acid [186]. Similarly, PdNPs were also electrodeposited on an epoxy-silver electrode, where the PdNPs act as catalyst for the direct oxidation of glucose [187]. Well-dispersed PdNPs were also prepared on graphene oxide (PdNPs/GO) using a simple ultrasonic method. The results showed that GO acted as a good supportive substrate for controlling the size and activity of PdNPs [6]. PdNPs deposited on surfactant-functionalized multi-walled carbon nanotubes (MWCNTs) were also synthesized by a facile spontaneous redox method. The as-prepared Pd catalyst showed excellent catalytic activity toward oxidation of ethanol and glucose which indicated a great potential for improving the performance of direct ethanol fuel cells and glucose sensors [188]. Furthermore, PdNPs supported on multi-walled carbon nanotubes (MWCNTs) were also synthesized by a simple *in situ* ultrasonication process at room temperature. The PdNPs could help increase the current signal due to their high surface area and the physical adsorption of the glucose molecules onto the large surface area of the electrode. More importantly, the electrode is highly resistant against poisoning by the interference from the oxidation of common interfering agents [189].

#### 3.12.2. Colorimetric and Spectrophotometric Detection

**Figure 5:**
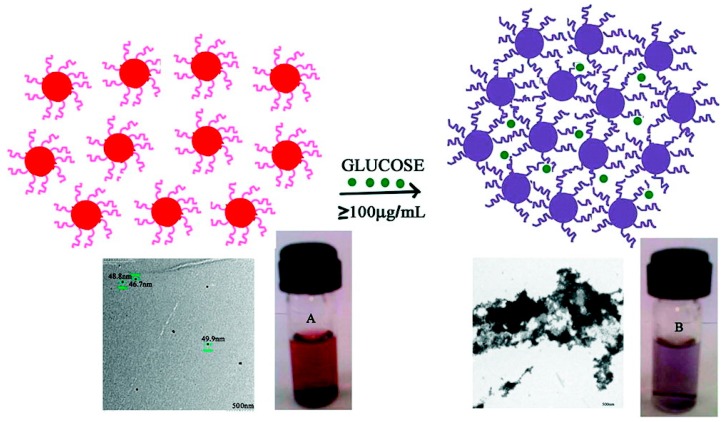
Color of (**A**) GNPs and (**B**) GOD-GNP on reacting with ≥100 μg/mL glucose, with mechanism [190]. Reprinted with permission from (Radhakumary, C.; Sreenivasan, K. Naked eye detection of glucose in urine using glucose oxidase immobilized gold nanoparticles. *Anal. Chem.* **2011**, *83*, 2829–2833.). Copyright (2011) American Chemical Society.

Colloidal gold is extensively used for molecular sensing because of the flexibilities it offers in terms of modification of the gold nanoparticles (AuNPs) surface with a variety of functional groups. To enable glucose detection, the naked eye detection of glucose in urine has been reported. Thiol-capped AuNPs were functionalized with glucose oxidase (GOD) using carbodiimide chemistry. A visible color change of the GOD-functionalized AuNPs from red to blue was observed [190], as shown in Figure 5. Another colorimetric assay for the detection of sugars was also demonstrated. The synthesis of Au-NPs in presence of glucose as reducing agent in different conditions has been achieved, allowing the formation of pink or blue color NPs, and this has been employed in the design of two colorimetric assays. Both assays relay on the analyte-induced intensity increase (without any shift) of the NPs plasmon band absorption. The pink assay is based on the sugar-assisted chemical synthesis of NPs while the other is based on the AuNPs synthesis catalyzed by the glucose oxidase enzyme. This colorimetric assay did not suffer from bleaching of the final color because the stability of the AuNPs [191].

A fluorometric method for the determination of glucose and hydrogen peroxide using BiFeO_3_ magnetic nanoparticles (BFO MNPs) has been reported. The authors found that BFO MNPs can catalyze the decomposition of H_2_O_2_ to produce OH radicals, which in turn oxidize the weakly fluorescent benzoic acid to a strongly fluorescent hydroxylated product with a maximum emission at 405 nm [192]. Moreover, a renewable glucose biosensor based on GOD immobilized on MNPs was constructed. The GOD was covalently cross-linked to the surface of synthesized Fe_3_O_4_ nanoparticles, then adhered to a solid parafin carbon paste electrode by magnetic force to fabricate a working electrode. H_2_O_2_ was produced by the enzymatic reaction of GOD and electrochemiluminescence (ECL) could be obtained by the reaction between luminol and H_2_O_2_ [193]. Furthermore, a colorimetric detection of glucose based MFe_2_O_4_ (M = Mg, Ni, Cu) MNPs has been proposed. This nanomaterial exhibited catalytic activities similar to those of biological enzymes that could catalyze H_2_O_2_ to produce hydroxyl radicals, which oxidized peroxidase substrate to produce a color [194].

Colorimetric detection based on assemblies of 5-amino-2-fluorophenylboronic acid-modified silver nanoparticles (FPBA-AgNPs) has been demonstrated. The glucose-modulated assembly of the FPBA-AgNPs occurred by the regulable formation of interparticle linkages via the bridge binding of 1,2-*cis*-diols and 5,6-*cis*-diols (for the furanose form) or 4,6-*cis*-diols (for the pyranose form), respectively, of a glucose molecule to two FPBA-AgNPs. Furthermore, the glucose level variations associated with a model biological reaction process can be monitored by using FPBA-AgNPs, whilst the reaction mechanism remains nearly unchanged [195]. A new assembled glucose sensor based on the AgNPs-enhanced fluorescence of CdSe quantum dots was also demonstrated. Compared to that of bare CdSe QDs, a fluorescence signal enhancement and a clear blue shift of the emission peak for AgNP-CdSe QD complexes were observed, which is attributed to the surface plasmon resonance of AgNPs. In addition, the as-formed complexes are gradually disassembled in the presence of glucose molecules because they can replace the AgNPs by competitive binding with boronic acid groups, resulting in the weakening of the fluorescence enhancement [196].

## 4. Analytical Performance

Analytical performance of selected samples are listed in tables that catagorized according to their detection methods including electrochemical (Table 1), and colorimetric and spectrophotometric techniques (Table 2), respectively.

**Table 1:** Selected examples of recent reports on biomedical targets using various nanoparticles by electrochemical techniques.

Group of Analytes	Detection *	Materials	Analytes	Detection Limit	Linear Dynamic Range	Ref.
Amino acids	Amperometry	AuNP-CNT/GCE	Trpysin	10 nM	30 nM–2.5 μM	[23]
	CV	Fe_3_O_4_-GO/GCE	Cysteine	56 μM	0.5–13.5 mM	[24]
			Acetaminophen	25 μM	0.12–13.3 mM	
	DPV	AgNPs/GO/GCE	Trpysin	2.0 nM	0.01–50 μM	[25]
					50.0–800.0 μM	
	CA	GC/DNA/NiOxNPs/Os(III)-complex electrode	Cysteine	0.07 μM	up to 1000 μM	[21]
Antigens-antibodies	SV	AuNPs	CEA	0.12 pg·mL^−1^	0.5 pg·mL^−1^–0.5ng·mL^−1^	[39]
Antioxidants	SWV	NiO/MWCNT	Glutathione	0.006 μM	0.01–200 μM	[49]
		nanocomposite	Acetaminophen	0.5 μM	0.8–600 μM	
	CV	Fe_2_O_3_/RG nanocomposite	Ascorbic acid	0.543 μM	0.57–3.97 μM	[48]
	Amperometry	AgNPs/CMWCNT/PANI/Au electrode	Glutathoine	0.3 μM	0.3–3500 μM	[47]
Cancer biomarkers	-	AuNP	Cancer-related gene sequence	4 fM	10 fM–1 nM	[58]
	Voltammetry	Fe_3_O_4_ MNPs	Leukemia cells	10 cells	-	[59]
	DPV	CdTe QDs/CGE	DNA sequences of bladder cancer cells	6.435 × 10^−13^ M	1 × 10^−12^–1 × 10^−8^ M	[61]
Chemical substances	CV	Fe_3_O_4_-Au-NPs	Digoxin	0.05 ng·mL^−1^	0.5–5 ng·mL^−1^	[78]
	CV/DPV/LSV	Fe_3_O_4_/GCE	Nimesulide	1.3 × 10^−7^ M	2.6 × 10^−6^–1.0 × 10^−4^ M	[10]
	DPV	AgNP/MWCNTs-COOH/GCE	Adriamycin	1.7 × 10^−9^ M	8.2 × 10^−9^–19.0 × 10^−9^ M	[76]
Hormones	-	WS2/AuNPs/GCE	17β-estradiol	2 pM	0.1 pM–5 nM	[93]
	Amperometry	NiOPs/Nafion-MWCNTs/SPE	Insulin	6.1 nM	20–60 nM	[94]
Lipids	Amperometry	AuE/dithiol/AuNPs/MUA/ ChOx	Cholesterol	34.6 μM	0.04–0.22 mM	[97]
	CA	AgNPs/GCE	Cholesterol	0.99 mg·dL^−1^	3.9–773.4 mg·dL^−1^	[99]
Microorganisms	EIS	EDC/NHS activated Au/1,6-HDT/AuNP/MUA	Type 5 adenovirus	30 virus particle·mL^−1^	10–10^8^ virus particle·mL^−1^	[102]
Nurotransmitters	DPV	AuNP-CNTs/PGE	l-Dopa	50 nM	0.1–150 μM	[107]
	DPV	NiFe_2_O_4_-MWCNT modified GCE	Dopamine	0.02 μM	0.05–6.0 μM 6.0–100 μM	[110]
	CV/LSV	RGO-Pd-NPs composite modified GCEs	Dopamine	0.233 μM	1–150 μM	[106]
	CV/DPA	PPyox-PTSA/Ag NP/Pt electrode	Dopamine	0.58 nM	1 × 10^−9^–1.2 × 10^−7^ M	[116]
Nucleic acids	DPV	AuNPs-ATP-diazo-ATP/Au electrode	ssDNA	9.10 × 10^−11^ M	3.01× 10^−10^–1.32 × 10^−8^ M	[127]
	Amperometry/CA/CV/DPV	Fe_3_O_4_/MWCNT/LDH/NAD^+^ modified GC electrode	NADH	0.3 μM	Up to 300 μM	[129]
	Amperometry/CV	Au nanoparticle/rGO GCE	NADH	1.13 nM	50 nM–500 μM	[131]
	CV	CdSe	Adenine Guanine	0.028 μM	0.083–291 μM	[132]
		QDs-GO		0.055 μM	0.167–245 μM	
Nucleic acids	DPV	AgNPs-Pdop@Gr /GCE	DNA	3.2 × 10^−15^ M	1 × 10^−13^–1 × 10^−8^ M	[134]
	CA	NiOxNPs/GC	NADH	106 nM	Up to 1 mM	[136]
Proteins	DPV	AuMNPs-Apt1/thrombin/Apt2-AuNPs-HRP modified Au electrode	Thrombin	30 fM	0.1–60 pM	[146]
	DPV	MNPs/CS-MWCNTs	Bovine serum albumin	2.8 × 10^−11^ g·mL^−1^	1.0 × 10^−4^–1.0 × 10^−10^ g·mL^−1^	[7]
Sugars	CA	AuNPs/GONRs/CS	Glucose	0.5 μM	2 μM–1.375 mM	[162]
					1.375–15 mM	
	Amperometry	MSENs/MGCE	Glucose	0.2 μM	0.5 μM–3.5 mM	[9]
	Amperometry	PVP-GNs-NiNPs-CS	Glucose	30 nM	0.1 μM–0.5 mM	[171]
	Amperometry	GOD-CS/AgNWs/ GCE	Glucose	2.83 μM	10 μM–0.8 mM	[177]
	Amperometry	CONM/GC	Glucose	0.15 μM	0.7–60 μM	[179]
	Amperometry	RGO-NiNPs/GCE	Glucose	0.1 μM	2 μM–2.1 mM	[184]
	Amperometry	Pd-MWCNTs	Glucose	0.2 μM	1–22 mM	[189]

* Detection methods were; (CA) Chronoamperometry, (CV) Cyclic Voltammetry, (DPV) Differential Pulse Voltammetry, (EIS) Electrochemical Impedance Spectroscopy (LSV) Linear Sweep Voltammetry, (SWV) Square Wave Voltammetry.

**Table 2:** Selected examples of recent reports on biomedical targets using various nanoparticles by colorimetric and spectrophotometric techniques.

Group of Analytes	Detections *	Materials	Analytes	Detection Limit	Linear Dynamic Range	Ref.
Amino acids	UV-Vis	CMC-AuNPs	Cysteine	ND	10.0–100.0 μM	[27]
	UV-Vis	Non-fluorosurfactant capped AgNPs	Cysteine	0.05 μM	1.5–6.0 μM	[16]
	SERS	AuNPs	Rabbit IgG	1–10 ng·mL^−1^	0–100 ng·mL^−1^	[40]
Antigens-antibodies	SPR	Fe_3_O_4_/SiO_2_ and Fe_3_O_4_/Ag/SiO_2_ MNPs	Rabbit IgG	ND	1.25–20 μg·mL^−1^ (for Fe_3_O_4_/SiO_2_)	[5]
					0.3–20 μg·mL^−1^ (for Fe_3_O_4_/Ag/SiO_2_)	
	ECL	Ag/graphene	CEA	0.6 pg·mL^−1^	1 pg·mL^−1^–500 ng·mL^−1^	[41]
	ECL	Si/CdTe/Ab_2_	Rabbit IgG	1.3 pg·mL^−1^	5 pg·mL^−1^–10 ng·mL^−1^	[42]
	ECL	AgNPs-rGO-Ab_2_-GOD	CEA	0.03 pg·mL^−1^	0.1 pg·mL^−1^–160 ng·mL^−1^	[43]
	Fluorescence	CdS-2MPA	Rutin	1.2 × 10^−6^ M	up to 4 × 10^−5^ M	[54]
Antioxidants	Spectrophotometry	BSA-AgNCs	Ascorbic acid	0.16 μM (3δ)	2.0–50.0 μM	[55]
	Colorimetric	Primer conjugated AuNPs	Human telomerase activity	1 Hela cell·μL^−1^	ND	[62]
Cancer biomarkers	Colorimetric	Fe_3_O_4_ MNPs and PtNPs nanohybrids	Target cancer cells (breast cancer)	ND	ND	[67]
	Wavelength-resolved imaging	Multiplexed QDs	Tumor cells in Hodgkin’s lymphoma	ND	ND	[69]
Chemical substances	CL	l-cysteine capped CdS QDs	Baclofen	0.0035 mg·L^−1^	0.012–24.0 mg·L^−1^	[89]
Hormones	SPR	AuNPs-G4-OH SAM	Insulin	0.5 pM	2–43 pM	[95]
	Fluorescence	Eu(III) chelated-bonded SiNPs	Human thyroid stimulating hormone	0.0007 mL·UL^−1^	0.005–100 ml·UL^−1^	[96]
Lipids	ECL	AuNPs	Cholesterol	5.7 nM	0.17 nM–0.3 mM	[100]
	ECL	Fe_3_O_4_@SiO_2_-Au@mpSiO_2_	Cholesterol	0.28 μM	0.83–2.62 mM	[101]
Microorganisms	CL	CMG-MNPs	HBV	0.5 pM	ND	[103]
	Fluorescence	Dual color QDs	Human enterovirus 71	0.42 ng·mL^−1^ (for EV71)	1–14 ng·mL^−1^ (For EV 71)	[105]
				0.39 ng·mL^−1^ (for CVB3)	1–19 ng·mL^−1^ (for CVB3)	
Neurotransmitters	Fluorescence	AgNPs	6-thioguanine	9.7 nM	1.5 × 10^−8^–7.5 × 10^−7^ M	[105]
	Spectrophotometry	AuNPs	Dopamine	33 nM	33 nM–3.33 mM	[123]
	UV-Vis	Co_x_Fe_3-x_O_4_ MNPs	Dopamine	0.13 μM	0.6–8.0 μM	[125]
Nucleic acids	Colorimetric in flow strip	Oligonucleotides-modified AuNPs	miRNA	1 fM (without silver enhancement)	ND	[140]
	Fluorescence	NEase-amplified MNPs	p53 gene	198 fM	ND	[142]
	CL	Oppy-PdNPs/Au	Sequence-specific DNA	6.0 × 10^−17^ M	1.0 × 10^−16^–1.0 × 10^−15^ M	[196]
Proteins	Colorimetric	Fib-AuNPs	Thrombin	ND	0.1–10 pM	[152]
	UV-Vis	Fe_3_O_4_@AuNPs	Thrombin	1.0 nM	1.6–30.4 nM	[155]
Proteins	Fluorescence	QD-apt nanoconjugates	Thrombin	1 nM	nM–μM	[158]
	Naked eyes	Apt-AgNPs	PDGF-BB	1.56 ng·mL^−1^	1.56 ng·mL^−1^–100 ng·mL^−1^	[29]
Sugars	Colorimetric	AuNPs	Glucose	10 μM (for pink assay)	Extended to 1.5 mM (for pink assay)	[191]
				5 μM (for blue assay)	Extended to 1.0 mM (for blue assay)	
	Fluorescence	BiFeO_3_ MNPs	Glucose	4.5 nM	0.2 nM–0.2 μM	[193]
	Colorimetric	FPBA-AgNPs	Glucose	89 μM	ND	[195]

* Detections were; (CL) Chemiluminescence, (ECL) Electrochemiluminescence, (SERS) Surface Enhanced Raman Scattering, (SPR) Surface Plasmon Resonance, (UV-Vis) UV-visible spectrophotometry.

## 5. Conclusions

Due to the good properties of inorganic nanoparticles, they have been prevalently used as powerful sensors and probes. Each type of nanoparticle exhibits its own fascinating properties. However, they shared a similarity of enhancing probe sensitivity owing to their high surface area-to-volume ratios. Metal particles mostly synthesized from their bulk material display remarkably properties and exceptional features due to their miniature size, while the excellent behavior of semiconductor and nanocomposites results from their combinations of various materials. They have been utilized in a variety of applications for numerous analytes as described in this review for both electrochemical and optical spectrophotometric detection. The use of nanoparticles is an excellent approach for biomedical detection. Various groups of examples as discussed clarify the efficiency of nanoparticles by an increment of signal. In addition to modification, nanoparticles are capable of providing selectivity against interferences and complicated matrix effects. Many benefits of nanoparticles have been revealed, although more feasible practices still need to be developed to overcome difficulties such as the tendency to agglomeration and precision in size-control. Moreover, differences in the synthesized products from day to day led to uncertain properties which stimulate the search for solutions. Applications of nanoparticles in biomedical detection are abundant as well. Among essential substances, small clusters such as single molecules or cells as early stage disease markers are recently of interest. Therefore, improved comprehension of the advantages resulting from the use of nanoparticles and curiosity is challenging many research groups and should lead to novel discoveries in the future.
